# Synthesis and Optical and Nonlinear Optical Properties of Linear and Two-Dimensional Charge Transfer Chromophores Based on Polyoxometalates

**DOI:** 10.1021/acs.inorgchem.4c04179

**Published:** 2024-12-06

**Authors:** Bethany R. Hood, Yovan de Coene, Claire F. Jones, Ivan Lopez Poves, Noah Deveaux, Nathan R. Halcovitch, Benoît Champagne, Koen Clays, John Fielden

**Affiliations:** † Department of Chemistry, Lancaster University, Lancaster LA1 4YB, United Kingdom; ‡ School of Chemistry, Pharmacy and Pharmacology, University of East Anglia, Norwich NR4 7TJ, United Kingdom; § Department of Chemistry, University of Leuven, Celestijnenlaan 200D, Leuven 3001, Belgium; ∥ Unit of Theoretical and Structural Physical Chemistry, Namur Institute of Structured Matter, University of Namur, Namur B-5000, Belgium; ¶ School of Chemistry, University of East Anglia, Norwich NR4 7TJ, United Kingdom

## Abstract

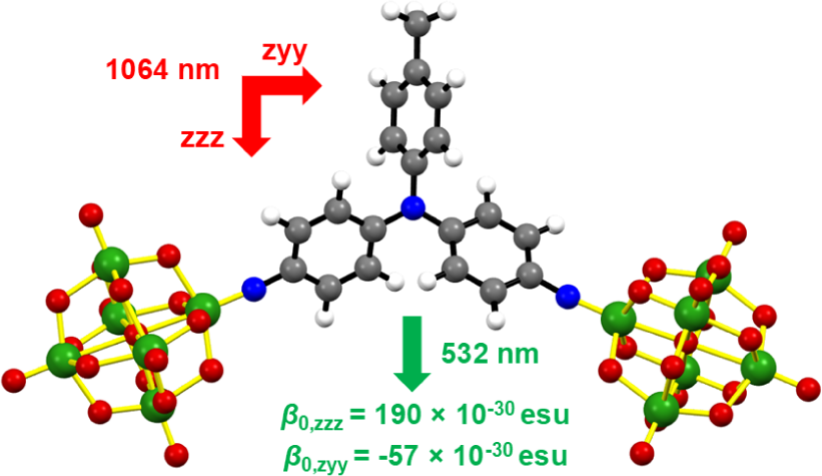

We present the first study of arylimido-polyoxometalate nonlinear optical (NLO) chromophores with two-dimensional (2D) structures, and a comparison with one-dimensional analogues, through the synthesis of a family of arylimido-hexamolybdate derivatives where one or two polyoxometalate (POM) acceptors are connected to a tolyl-amino donor through phenyl bridges. Electronic absorption spectra and TD-DFT calculations reveal significant red-shifts in ligand-to-polyoxometalate charge transfer (LPCT) absorption bands for the 2D species compared to linear, dipolar analogues, consistent with the involvement of a larger conjugated (bridge + POM) system in the transitions. Electrochemical measurements indicate reversible, one-electron processes for the POM acceptors with class II mixed valence behavior observed where the POMs are connected to the same aryl ring and electronic isolation of the acceptors when they are on separate rings. Molecular first hyperpolarizabilities β have been determined using hyper-Rayleigh scattering at 1064 and 1200 nm: for the most active compound, the HRS measurements and depolarization studies reveal a strongly 2D, off-diagonal response (β_0,zzz_ = 190 × 10^–30^ esu; β_0,zyy_ = −56.5 × 10^–30^ esu), consistent with the wide A–D–A angle and TD-DFT computed electronic transitions, which show both phenyl bridges and POMs equally involved in the acceptor orbitals.

## Abstract 2

Hybrid arylimido-polyoxometalate (POM) acceptor−donor−acceptor charge transfer chromophores, where the POM acts as an acceptor, can show strong and highly two-dimensional nonlinear optical responses. The best performance (β_0,zzz_ = 190 × 10^−30^ esu; β_0,zyy_ = −56.5 × 10^−30^ esu) arises when the POMs are each separated from the donor by an independent phenyl bridge.

## Introduction

Polyoxometalates (POMs) are a family of well-defined anionic metal oxide cluster compounds, typically based on Mo, W, or V, whose wide range of structures, sizes, shapes, and elemental compositions give rise to an equally broad array of properties. For example, superacidity and stable redox chemistry have long given POMs applications in catalysis, and the inclusion of heterometal sites or the connection of organic groups to the POM framework can introduce catalytic active sites and magnetic or optical properties.^1^ By connecting POMs to conjugated organic systems with strong electronic communication, new electronic and optical properties can emerge: we have been focused on the nonlinear optical (NLO) properties of arylimido POMs (a-POMs) that arise from strong POM/aryl electronic coupling.^2^ The nonlinear optical response of molecular chromophores allows for the manipulation of laser light with faster and more tunable responses than those seen in the inorganic salts currently in use, leading to potential uses in areas such as telecommunications, optical/electro-optical computing, and imaging.^3^

These hybrid charge transfer (CT) chromophores, compared to typical purely organic and other metallo-organic systems, show a promising combination of high second-order nonlinear optical activity with short, thermally stable π-bridges and high visible transparency—a challenge for molecular chromophores, as design features that tend to increase the nonlinear optical response (β), i.e., stronger donor–acceptor pairs and extended π-systems, also shift absorptions further into the visible and near-infrared. This leads to undesirable reabsorption of visible light, lowering efficiency and stability in real applications. The polyoxometalate acceptors are also capable of redox-switched responses,^4^ a potential basis for electro-optical computing or data storage.

Transparency and nonlinearity trade-offs have been addressed in other classes of organic and metallo-organic NLO chromophores through compounds with unusual donor/acceptor systems and innovative bridges.^5^ In addition, there has been a focus on multidimensional chromophores such as compounds with octupolar, *C*_*2v*_ (V-shaped), or X-shaped symmetry, rather than the conventional 1D dipolar charge transfer architecture.^6^ In many cases, such multidimensional systems provide enhanced NLO responses (*β)* without the loss of transparency associated with tailoring the donors, acceptors, and bridges of a 1D charge-transfer system. Moreover, in V-shaped species, the off-diagonal response—that is, the presence of more than one significant component of β—prevents reabsorption of harmonic light because the CT transition dipole moment (μ) is perpendicular to the dipole moment change (*Δμ*) resulting from this excitation and also facilitates phase matching between the fundamental and harmonic waves when applied in materials for second harmonic generation (SHG).^7^ Multidimensional a-POM derivatives are accessible by either attaching two a-POM acceptor units via π-bridges to a donor to produce [(Mo_6_O_18_N)_2_ArD]^4–^ compounds (ArD = aryl bridge and donor) or by synthesis of [Mo_6_O_17_(NArD)_2_]^2–^*bis*-derivatized POM cores,^8^ but there have been no experimental studies of second-order NLO properties. Prior computational work on D–A–D systems of the latter [Mo_6_O_17_(NArD)_2_]^2–^ type suggested high NLO activities and large off-diagonal components,^9^ but such gas phase calculations using GGA functionals have since been found to poorly reproduce experimental results for other a-POM systems.^2b^ Thus, to date, the second-order NLO properties of multidimensional POM-based chromophores have not been satisfactorily addressed.

Recently, we investigated the third-order NLO properties (two photon absorption) of compounds with [(Mo_6_O_18_N)_2_Ar]^4–^ geometry (Ar = aryl bridge, no donor) with both linear centrosymmetric and *C*_*2v*_ symmetry.^10^ Lacking donor groups, these compounds have insufficient dipolar charge transfer character for appreciable second-order (β) responses. Herein, we introduce tolyl amine donors to a related series of two-dimensional a-POM chromophores and assess their NLO activity experimentally through hyper-Rayleigh Scattering and computationally by TD-DFT using methods (range-separated hybrid functional, with solvation) that have proved reliable for such species.^2d,2e^ The results reveal that such compounds can have substantial, strongly two-dimensional β responses with large off-diagonal components.

## Experimental Section

### Materials and Procedures

Dry dimethyl sulfoxide (DMSO) was purchased from Sigma-Aldrich (SureSeal) and Acros Organics (Acro Seal) and used as supplied. All other reagents and solvents were obtained as ACS grade from Sigma-Aldrich, Alfa Aesar, Fisher Scientific, Fluorochem, Acros Organics, or Apollo Scientific and used as supplied. Deuterated solvents were obtained from Eurisotop, Cambridge Isotope Laboratories, or Acros Organics and used as supplied. Tetrabutylammonium hexamolybdate^11^ was synthesized according to previously published methods. Cellulose powder for column chromatography and cellulose TLC plates were purchased from Merck. Organic precursors (**P1** to **P8**) to the arylimido-polyoxometalates described later were synthesized using adaptations of previously reported methods, with full details given in the Supporting Information. The functionalization of hexamolybdate was adapted from known procedures.^12^ All reactions were performed under an atmosphere of dry argon by using standard Schlenk techniques. Syntheses of the starting anilines and other precursors are described in the Supporting Information.

### General Physical Measurements

FT-IR spectra were measured using a Bruker FT-IR XSA spectrometer. ^1^H- and ^13^C NMR spectra were acquired using a Bruker Ascend 500 (500 MHz) spectrometer, and all shifts are quoted with respect to TMS using the solvent signals as secondary standards (s = singlet, d = doublet, *t* = triplet, q = quartet, quin = quintet, sex = sextet, *a*sex = apparent sextet, hept = heptet, and m = multiplet). Some quaternary carbon signals were not observed for [NBu_4_]_2_[**4**], similar to previously synthesized arylimido-polyoxometalates.^2^ Elemental analyses and accurate mass spectrometry were outsourced to the University of Manchester and the John Innes Centre (Norwich) or UK National Mass Spectrometry Service (Swansea), respectively. Note that for polyoxometalate samples, the strength of the monoisotopic ^92^Mo peaks is too weak to match, but theoretical isotope profiles are excellent matches to observed peak envelopes, and matches for individual peaks within the envelope are well within required 5 ppm errors. UV–vis spectra were obtained using an Agilent Cary 60 UV–vis spectrophotometer.

### Preparation of [NBu_4_]_4_[Mo_12_O_36_ N_3_C_19_H_15_] ([NBu_4_]_4_[**1**])

A solution of tetrabutylammonium hexamolybdate (1.305 g, 0.976 mmol), 4,4′-diamino-4″-methyltriphenylamine (precursor **P2**, 0.118 g, 0.373 mmol), and DCC (0.230 g, 1.11 mmol) in 15 mL of dry dimethyl sulfoxide was heated at 70 °C for 10 h. Once cool, the mixture was filtered to remove the pale precipitate and then poured into 40 mL of ethanol and 150 mL of diethyl ether to give a dark precipitate. The precipitate was washed with ethanol and diethyl ether before being collected by filtration to give crude [NBu_4_]_4_[**1**] (1.225 g). ^1^H NMR indicated this material contained *ca*. 30% unreacted hexamolybdate, and further purification was achieved by washing with DCM before reprecipitating a concentrated acetone solution using diethyl ether to give the pure compound [NBu_4_]_4_[**1**] (0.140 g, 0.050 mmol) in an overall 13% yield. ^1^H NMR (500 MHz, CD_3_CN) δ 7.20 (d, *J* = 7.20 Hz, 2H), 7.12 (d, *J* = 8.8 Hz, 4H), 7.01 (d, *J* = 8.4 Hz, 2H), 6.95 (d, *J* = 8.9 Hz, 4H), 3.18–3.04 (m, 32H), 2.35 (s, 3H), 1.64–1.58 (m, 32H), 1.36 (*a*sex, *J* = 7.4 Hz, 32H), 0.97 (t, *J* = 7.4 Hz, 48H). ^13^C NMR (126 MHz, CD_3_CN) δ: 151.0, 147.6, 136.4, 131.7, 131.3, 128.3, 127.5, 123.3, 59.2, 24.2, 20.8, 20.2, 13.8. Anal. (Calcd) % for C_83_H_159_Mo_12_O_36_N_7_·(CH_3_)_2_CO: C 34.10 (33.97); H 5.32 (5.47); N 3.45 (3.22). HRMS (ESI, MeCN) = calcd for C_19_H_15_N_3_Mo_12_O_36_^(4−)^ ([**1**]^4–^) 503.2044, found 503.2046. ATR: 2960 (m), 2932 (sh), 2872 (m), 2163 (w), 2034 (w), 1576 (m), 1508 (m), 1482 (s), 1379 (m), 1315 (s), 1286 (m), 1167 (m), 1106 (w), 1065 (w), 1029 (w), 973 (m), 943 (vs), 882 (sh), 764 (vs). UV–vis (MeCN) λ, nm (ε, M^–1^ cm^–1^): 200 (100.1 × 10^3^); 255 (59.2 × 10^3^); 337 (22.2 × 10^3^); 473 (49.4 × 10^3^).

### Preparation of [NBu_4_]_4_[1,3,5-Mo_12_O_36_N_3_C_20_H_17_] ([NBu_4_]_4_[**2**])

A solution of tetrabutylammonium hexamolybdate (0.859 g, 0.656 mmol), 3,5-diamino-4′,4′′-dimethyltriphenylamine (precursor **P4**, 0.094 g, 0.310 mmol), and DCC (0.157 g, 0.761 mmol) in 15 mL of dry dimethyl sulfoxide was heated at 70 °C for 10 h. Once cool, the solution was filtered to remove the pale precipitate and then poured into 40 mL of ethanol and 150 mL of diethyl ether to give a dark precipitate. The precipitate was washed with ethanol and diethyl ether before being collected by filtration to give crude [NBu_4_]_4_[**2**] (0.648 g) as a red solid. ^1^H NMR indicated this contained close to 50% unreacted hexamolybdate, so purification of 204 mg was achieved by slow recrystallization in acetone/diethyl ether to give pure [NBu_4_]_4_[**2**] (0.067 g, 0.022 mmol) in an overall 23% yield. ^1^H NMR (500 MHz, CD_3_CN) δ 7.21 (dt, *J* = 7.3, 0.8 Hz, 4H), 7.03 (d, *J* = 8.3 Hz, 4H), 6.41 (t, *J* = 1.7 Hz, 1H), 6.29 (d, *J* = 1.7 Hz, 2H), 3.18–3.09 (m, 32H), 2.34 (s, 6H), 1.67–1.59 (m, 32H), 1.38 (*a*sex, *J* = 7.4 Hz, 32H), 0.97 (t, *J* = 7.4 Hz, 48H). ^13^C NMR (126 MHz, CD_3_CN) δ: 155.0, 144.8, 135.9, 131.3, 126.8, 116.3, 116.1, 115.3, 59.2, 24.2, 20.9, 20.2, 13.7. Anal. (Calcd) % for C_84_H_161_Mo_12_O_36_N_7_·(CH_3_)_2_CO: C 34.15 (34.21); H, 5.45 (5.51); N, 3.26 (3.21). HRMS (ESI, MeCN) = calcd for C_52_H_89_N_5_Mo_12_O_36_^(2-)^ (**[2**+2NBu_4_]^2–^), 755.0391, found 755.0385. ATR: 2961 (m), 2933 (sh), 2873 (m), 1708 (m), 1559 (m), 1507 (m), 1460 (m), 1425 (m), 1379 (m), 1362 (sh), 1309 (sh), 1293 (sh), 1258 (m), 1222 (sh), 1158 (w), 1108 (w), 1058 (w), 1033 (w), 975 (m), 944 (s), 764 (vs), 737 (vs). UV–vis (MeCN) λ, nm (ε, M^–1^ cm^–1^): 257 (24.0 × 10^3^); 275 (24.8 × 10^3^); 345 (16.5 × 10^3^); 469 (1.65 × 10^3^)

### Preparation of [NBu_4_]_4_[1,2,4-Mo_12_O_36_N_3_C_20_H_17_] ([NBu_4_]_4_[**3**])

A solution of tetrabutylammonium hexamolybdate (0.418 g, 0.306 mmol), 2,4-diamino-4′,4′′-dimethyltriphenylamine (precursor **P6**, 0.046 g, 0.152 mmol), and DCC (0.077 g, 0.373 mmol) in 15 mL of dry dimethyl sulfoxide was heated at 70 °C for 10 h. Once cool, the solution was filtered to remove the pale precipitate and then poured into 40 mL of ethanol and 150 mL of diethyl ether to give a dark precipitate. The precipitate was washed with ethanol and diethyl ether before being collected by filtration to give crude [NBu_4_]_4_[**3**] (0.393 g, 0.131 mmol) as a red solid in a 35% yield. ^1^H NMR indicated the presence of small amounts of aniline and hexamolybdate starting materials, so further purification of 201 mg of the crude material was achieved by crystallization in acetone/diethyl ether vapor diffusion to give compound [NBu_4_]_4_[**3**] (0.065 g, 0.022 mmol) in an overall 11% yield. ^1^H NMR (500 MHz, CD_3_CN) δ 7.09 (d, *J* = 8.0 Hz, 4H), 6.93 (d, *J* = 0.6 Hz, 1H), 6.92 (d, *J* = 2.1 Hz, 1H), 6.88 (dd, *J* = 2.1, 0.6 Hz, 1H), 6.84 (d, *J* = 8.4 Hz, 4H), 3.30–3.09 (m, 32H), 2.34 (s, 6H), 1.73–1.58 (m, 32H), 1.39 (*a*sex, *J* = 7.3 Hz, 32H), 0.97 (t, *J* = 7.3 Hz, 48H). ^13^C NMR (126 MHz, CD_3_CN) δ: 150.7, 145.4, 144.3, 134.6, 130.7, 128.0, 127.3, 127.1, 126.7, 124.5, 59.2, 24.3, 20.9, 20.2, 13.7. Anal. (Calcd) % for C_84_H_161_Mo_12_O_36_N_7_: C 33.98 (33.67); H, 5.50 (5.42); N, 3.30 (3.27). HRMS (ESI, MeCN) = calcd for C_52_H_89_N_5_Mo_12_O_36_^(2−)^ (**[3**+2NBu_4_]^2–^), 755.3725, found 755.3728. ATR: 2961 (m), 2934 (sh), 2873 (m), 1731 (w), 1608 (w), 1567 (w), 1485 (m), 1469 (m), 1379 (m), 1348 (w), 1296 (m), 1259 (sh), 1152 (w), 1108 (w), 1065 (w), 1025 (w), 974 (m), 944 (s), 879 (w), 767 (vs). UV–vis (MeCN) λ, nm (ε, M^–1^ cm^–1^): 257 (58.5 × 10^3^); 262 (58.7 × 10^3^); 340 (33.1 × 10^3^); 461 (26.4 × 10^3^).

### Preparation of [NBu_4_]_2_[Mo_6_O_18_N_2_C_20_H_18_] ([NBu_4_]_2_[**4**])

A solution of tetrabutylammonium hexamolybdate (0.688 g, 0.50 mmol), 4-amino-4′,4′′-dimethyltriphenylamine (precursor **P8**, 0.134 g, 0.46 mmol), and DCC (0.150 g, 0.73 mmol) in dry dimethyl sulfoxide (10 mL) was heated at 70 °C for 10 h, with a color change to dark brown in the first 15 min. The reaction mixture was cooled to room temperature, and the solution was filtered into a mixture of 40 mL of ethanol and 160 mL of diethyl ether to give a highly viscous dark red oil. This was washed with the Et_2_O/EtOH mixture (3 × 15 mL) to yield crude [NBu_4_]_2_[**4**] (0.320 g, 0.20 mmol, 43%). A portion (0.12 g) was removed and crystallized for X-ray diffraction, and the remaining 0.20 g was purified by flash chromatography on a cellulose-packed column (25 g) using toluene 5:1 acetonitrile as eluent, leaving unreacted hexamolybdate on the column. Collection of fractions containing a red spot with *R*_f_ = 0.74 and removal of solvent *in vacuo* produced a red oil, which was triturated with Et_2_O (3 × 3 mL) to remove residual unreacted amine precursor, yielding [NBu_4_]_2_[**4**] as a dark red powder (0.052 g, 0.032 mmol, 11% overall allowing for removal of crystallized portion). ^1^H NMR (400 MHz, CH_3_CN) δ: 7.16 (d, *J* = 8.2 Hz, 4H), 7.06 (d, *J* = 8.9 Hz, 2H), 6.99 (d, *J* = 8.2 Hz, 4H) 6.77 (d, *J* = 8.9 Hz, 2H), 3.11 (m, 16H), 2.32 (s, 6H) 1.61 (m, 16H) 1.37 (*a*sex, *J* = 7.5 Hz, 16H), 0.97 (t, *J* = 7.3 Hz, 24H). ^13^C NMR (126 MHz, CD_3_CN) δ: 145.0, 145.5, 131.2, 128.5, 126.9, 126.0, 120.0, 59.4, 24.4, 20.9, 20.4, 13.8. Anal. (Calcd) % for C_52_H_90_Mo_6_N_4_O_18_: C 38.22 (38.20); H, 5.20 (5.55); N, 3.42 (3.43). HRMS (ESI, MeCN) = calcd for C_20_H_18_N_2_Mo_6_O_18_^(2−)^ ([**4**]^2–^), 574.7457, found 574.7467. ATR: 2961 (m), 2933 (sh), 2873 (m), 1630 (w), 1607 (w), 1583 (m), 1506 (m), 1489 (m), 1469 (sh), 1379 (m), 1320 (m), 1295 (m), 1281 (sh), 1270 (sh), 1169 (w), 1107 (w), 1064 (vw), 1032 (vw), 1020 (sh), 974 (m), 946 (s), 882 (w), 769 (vs). UV–vis (MeCN) λ, nm (ε, M^–1^ cm^–1^): 201 (88.0 × 10^3^); 291 (28.5 × 10^3^); 430 (34.7 × 10^3^).

### Electrochemistry

Cyclic voltammetry and bulk electrolysis experiments were carried out by using an Autolab PGstat 30 potentiostat/galvanostat. Measurements were performed in a single-compartment cell using a silver wire reference electrode, a glassy carbon working electrode, and a platinum wire counter electrode. Acetonitrile was freshly distilled (from CaH_2_), and [N(C_4_H_9_-*n*)_4_]BF_4_^13^ was used as the supporting electrolyte. Solutions containing *ca*. 0.8 mM analyte (0.1 M electrolyte) were degassed by purging with argon and blanketed with a continuous flow of argon throughout the experiments. *E*_1/2_ values were calculated from (*E*_pa_ + *E*_pc_)/2 at a scan rate of 100 mV s^–1^ and referenced to Fc/Fc^+^.

### X-Ray Crystallography

X-ray quality crystals of [NBu_4_]_4_[**2**]·(H_3_C)_2_CO·H_2_O, [NBu_4_]_4_[**3**]·H_2_O, and [NBu_4_]_2_[**4**] were grown by diffusion of diethyl ether into acetonitrile, diffusion of diethyl ether into acetonitrile, and hot recrystallization from EtOAc 2:9 DCM, respectively. Multiple attempts to grow crystals of [NBu_4_]_2_[**1**] failed to produce suitable materials. Data were collected on a Rigaku XtalLab Synergy S diffractometer using a Photon-Jet Mo or Cu microfocus source and a Hypix hybrid photon counting detector. Data reduction, cell refinement, and absorption correction were carried out using Rigaku CrysAlisPro,^14^ and the structure was solved with SHELXT^15^ in Olex2 V1.5.^16^ Refinement was achieved by full-matrix least-squares on all *F*_0_^2^ data using SHELXL (v. 2018–3),^17^ also in Olex2 V1.5. Full crystallographic data and refinement details are presented in Table S1, and an ORTEP representation of the asymmetric units are provided in Figures S1–S3.

### Hyper-Rayleigh Scattering

General details of the hyper-Rayleigh scattering (HRS) experiment have been discussed elsewhere,^18^ and the experimental procedure and data analysis protocol used for the fs measurements in this study were as previously described.^19^ Measurements were carried out using dilute (ca. 10^–5^ M) filtered (Millipore, 0.45 μm) acetonitrile solutions such that self-absorption of the scattered second harmonic signal was negligible, verified by the linear relation between signal and concentration. The 1064 nm source was a Spectra-Physics InSight DS+ laser (1 W average power, sub-100 fs pulses, 80 MHz). The collection optics were coupled to a spectrograph (model Bruker 500is/sm), together with an EMCCD camera (Andor Solis model iXon Ultra 897). Correction for multiphoton-induced fluorescence was done by subtracting the broad MPF background signal from the narrow HRS peak (fwhm ±9 nm). The high accuracy of this setup enables us to use the solvent as an internal reference (acetonitrile, *β*_HRS,1064_ = 0.258 × 10^–30^ esu; *β*_*zzz*,1064_ = 0.623 × 10^–30^ esu).^20^ Depolarization ratios ρ were determined following established methods.^21^ β tensor components β_*zzz*_ and β_*zyy*_ were extracted by assuming Kleinman and planar symmetry for *C*_*2v*_ molecules, yielding only two significant components of the β tensor, β_*zzz*_ and β_*zyy*_. These are determined from orientationally averaged  and ρ by applying eqs 1–3:^21^
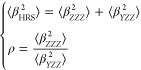
1

The HRS intensities with parallel polarization for fundamental and SH wavelengths, , and those for perpendicular polarization, , can be expressed in terms of the molecular components β_*zzz*_ and β_*zyy*_ as follows:
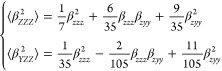
2

The depolarization ratio ρ can be expressed in terms of the ratio between the molecular β components, *k* = β_*zzz*_/β_*zyy*_.
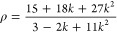
3

This approach can be applied to non-*C*_*2v*_ geometries, as seen, for example, for [NBu_4_]_4_[**3**], in which case it produces *effective* values of the tensor components that give an indication of the dimensionality of the β response.

### Quantum Chemical Calculations

Geometry optimizations were performed by density functional theory (DFT), using the range-separated ωB97X-D exchange-correlation functional (XCF)^22^ with 6-311G(d)^23^ (C, H, N, and O) and LANL2TZ^24^ (Mo) basis sets. Solvent effects (acetonitrile) were modeled using the integral equation formalism of the polarizable continuum model (IEF-PCM).^25^ The reliability of the ωB97X-D/6-311G(d)/LANL2TZ method for the geometry optimization of POM derivatives was demonstrated in comparison with other XC functionals in a previous work.^2e^ Excited state properties were calculated for the optimized geometries using time-dependent density functional theory (TD-DFT),^26^ with the same XCF, basis set, and IEF-PCM solvation. The 30 lowest excitation energies, oscillator strengths, and transition dipole moments *μ*_ge_ were calculated together with the ground-to-excited state dipole moment changes *Δμ*_ge_, charge transfer distances *d*_CT_, and amounts of charge transferred *q*_CT_, according to the scheme presented by Le Bahers et al.^27^ Again with the same optimized geometries, XCF, basis set, and IEF-PCM scheme, SHG β tensor components were evaluated using the quadratic response TD-DFT method:^28^ ωB97X-D has been shown to be a reliable XCF for calculating the β tensors owing to its substantial amount of long-range HF exchange.^29^ Both static and dynamic (incident wavelength of 1064 and 1200 nm) responses were calculated, and molecular responses have been analyzed by using the computed depolarization ratios and assuming Kleinman symmetry to obtain the two components β_*zzz*_ and β_*zyy*_, as described above for the HRS results. Further details of all computational aspects are provided in the SI.

## Results and Discussion

### Chromophore Design and Synthesis

**Figure 1:**
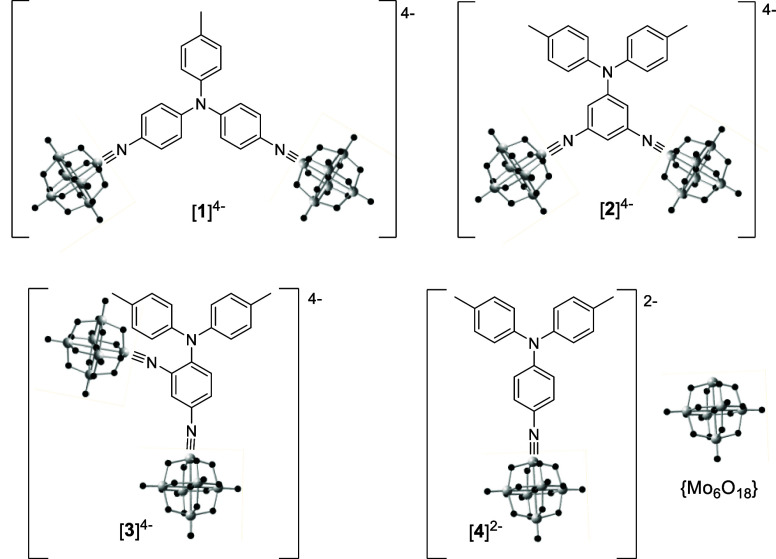
Chromophoric anions [**1**]^4–^ to [**4**]^2–^, in all cases isolated as tetrabutylammonium (NBu_4_^+^) salts.

To investigate the effects of different 2D donor/acceptor geometries, the series of tolyl or ditolyl donor, *bis*-POM anionic chromophores [**1**]^4–^, [**2**]^4–^, and [**3**]^4–^ were synthesized as tetrabutylammonium salts, with the ditolylamino donor system [**4**]^2–^ synthesized as a 1D comparison (Figure 1). In anion [**1**]^4–^, the two POM acceptor units are both connected to a shared tolylamine donor by their own *para*-phenyl bridge; in [**2**]^4–^, the POMs both connect to the same phenyl bridge with a *meta* relationship to the ditolylamine donor group, while [**3**]^4–^ is an isomer of [**2**]^4–^ with *para*- and *ortho-* donor–acceptor relationships to maximize the CT communication.

All four compounds were obtained from tetrabutylammonium hexamolybdate and the appropriate donor-functionalized triarylamine using previously reported DCC-mediated coupling protocols^2,11,12^ and characterized by ^1^H NMR (Figures S1–S4), ^13^C NMR, mass spectrometry (Figures S4–S8), IR and UV–vis spectroscopy, and CHN elemental analysis. To ensure functionalization of all aniline sites and to prevent the formation of *bis*-imido-{Mo_6_} species, reactions were carried out using excess [NBu_4_]_2_[Mo_6_O_19_], and initially isolated crude products typically contained substantial amounts (up to 50%) of unreacted hexamolybdate. This was removed from the *bis*-POM products [NBu_4_]_4_[**1**] to [**3**] by recrystallization or reprecipitation, at a high cost in yield (final pure yields 11–23%) due to the relatively similar solubilities of both [NBu_4_]_2_[Mo_6_O_19_] and the derivatives. For the *mono*-POM product [NBu_4_]_2_[**4**], [NBu_4_]_2_[Mo_6_O_19_] was removed by chromatography over cellulose; however, the final yield was still low. Generally, the removal of [NBu_4_]_2_[Mo_6_O_19_] from its arylimido derivatives is challenging due to similar solubilities and the susceptibility of the derivatives to hydrolysis.

Synthesis of the precursor anilines is fully described in the SI. For the precursors to [NBu_4_]_4_[**1**], [NBu_4_]_4_[**3**], and [NBu_4_]_2_[**4**], this was achieved by using a nucleophilic aromatic substitution procedure between tolylamine or ditolylamine and the appropriate nitro- or dinitro-fluorobenzene, followed by reduction of the nitro group(s).^2d,30^ Due to the 1,3,5 geometry lessening activation of the halogen leaving group to S_N_Ar reactions, the organic precursor for [NBu_4_]_4_[**2**] was synthesized using a Buchwald coupling from 1-bromo-3,5-dinitrobenzene and di-p-tolylamine, followed again by reduction of the nitro groups.

^1^H NMR analysis (Table 1) reveals a number of trends in the chemical shift consistent with the substitution patterns of the phenyl bridges. All of the tolyl Me resonances of the *bis*-POM derivatives [**1**]^4–^ to [**3**]^4–^ show a slight upfield shift compared to the *mono*-POM derivative [**4**]^2–^, consistent with the electron withdrawing effect of a second acceptor. Notably, the phenyl resonances of [**2**]^4–^, with a *meta* relationship between the imido-POM groups, show much less upfield shift than the other compounds. This is likely because the weaker donor/acceptor communication resulting from the 1,3 donor–acceptor geometry produces a less polar electronic structure, where the three phenyl protons have *ortho* or *para* relationships to the ditolylamino donor, as well as to the POM acceptors. Comparison of [**1**]^4–^ and [**4**]^2–^ reveals upfield chemical shifts upon replacement of a methyl group with a second POM, consistent with the known electron acceptor properties. Interestingly, despite improved donor/acceptor communication in [**3**]^4–^ compared to [**2**]^4–^, the aromatic tolyl protons of [**3**]^4–^ have smaller upfield chemical shifts than those of [**2**]^4–^. This may result from through space shielding of the tolyl group by interaction with the large and negatively charged *ortho* POM cluster.

**Table 1:**
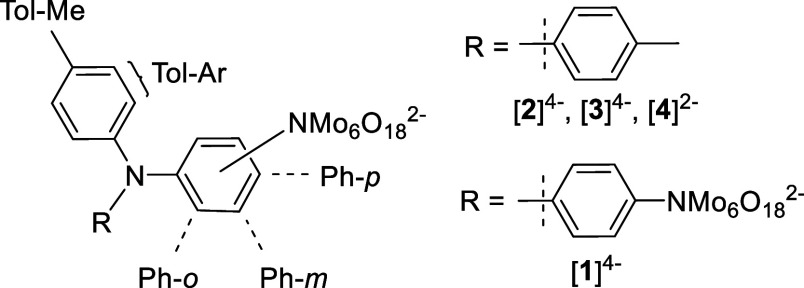
Shifts of Selected ^1^H-NMR Peaks of **1**, **2**, **3**, and **4** in Acetonitrile^a^

Anion	Tol-Me	Tol-Ar		Ph-*o*	Ph**-***m*	Ph**-***p*
[**1**]^4–^	2.35	7.20	7.01	6.95	7.12	-
[**2**]^4–^	2.34	7.21	7.03	6.29	-	6.41
[**3**]^4–^	2.34	7.09	6.84	6.88	6.93/6.92	-
[**4**]^2–^	2.32	7.16	6.99	6.78	7.05	-

a All spectra are referenced to TMS and reported in ppm.

### X-Ray Crystallography

**Figure 2:**
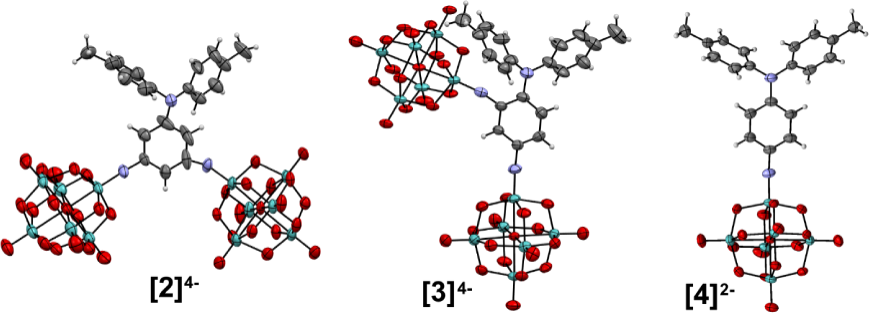
ORTEP representations of the structures of the anions in [NBu_4_]_4_[**2**], [NBu_4_]_4_[**3**], and [NBu_4_]_2_[**4**]. Thermal ellipsoids are drawn at the 30% probability level. Disorder in [**2**]^4–^ has been omitted for clarity but is shown in Figure S9. C is shown in gray, N in purple, O in red, and Mo in green. H atoms are represented by white spheres of arbitrary radii.

X-ray quality crystals of [NBu_4_]_4_[**2**]·(H_3_C)_2_CO·H_2_O, [NBu_4_]_4_[**3**]·H_2_O, and [NBu_4_]_2_[**4**] were obtained by ether diffusion or hot recrystallization, and structures were obtained (Figures 2 and S9–S11), but multiple attempts to grow [NBu_4_]_4_[**1**] produced only oils. Mo–O bond lengths of all three compounds were consistent with those of known [Mo_6_O_18_NAr]^2–^ clusters,^2,31^ as were imido Mo–N distances (Table S2). Significant variations in the Mo–N–C bond angle are observed, from 158.4° in one of the disordered parts of [**2**]^4–^ to a much more linear 175.6(8)° for the POM *para* to the donor in [**3**]^**4–**^. Similar variations have been noted in previous work,^2b^ and an absence of any clear pattern with the connected donor group suggests that this is driven by crystal packing effects, plus steric and other intramolecular interactions, rather than electronic differences—although, interestingly, the pattern of variations observed in [**2**]^4–^ to [**4**]^2–^ is quite well reproduced by computation (*vide infra*). As noted with other arylamine donor systems compared to alkylamino donors,^2c,2d^ there is little evidence of a quinoidal structure in the aryl bridges in these compounds, but the structure around the donor-N is consistent with some variations in donor–acceptor communication. Thus, in [**4**]^2–^, the donor N is almost perfectly trigonal planar, showing C–N–C bond angles between ca. 118° and 122° and effectively no (ca. 0.01 Å) displacement from the plane of the three connected carbons and indicating strong conjugation of the N lone pair with the π-bridge. In [**3**]^4–^, steric interactions with the POM in the 2-position cause some distortion away from trigonal planar, giving C–N–C bond angles between *ca*. 111° and 124° and a small displacement (ca. 0.20 Å) of the N from the plane of the three connected carbons, potentially weakening donor–acceptor communication. In [**2**]^4–^, disorder of the ditolylamino donor over two positions with roughly equal occupancies reveals one site with a similar, near trigonal-planar geometry to that of [**4**]^2–^ and another with near identical distortion to [**3**]^4–^. This is consistent with weaker donor–acceptor communication in the 1,3,5 system, enabling more flexibility in the geometry around the donor nitrogen.

### Electronic Spectroscopy and Electrochemistry

**Figure 3:**
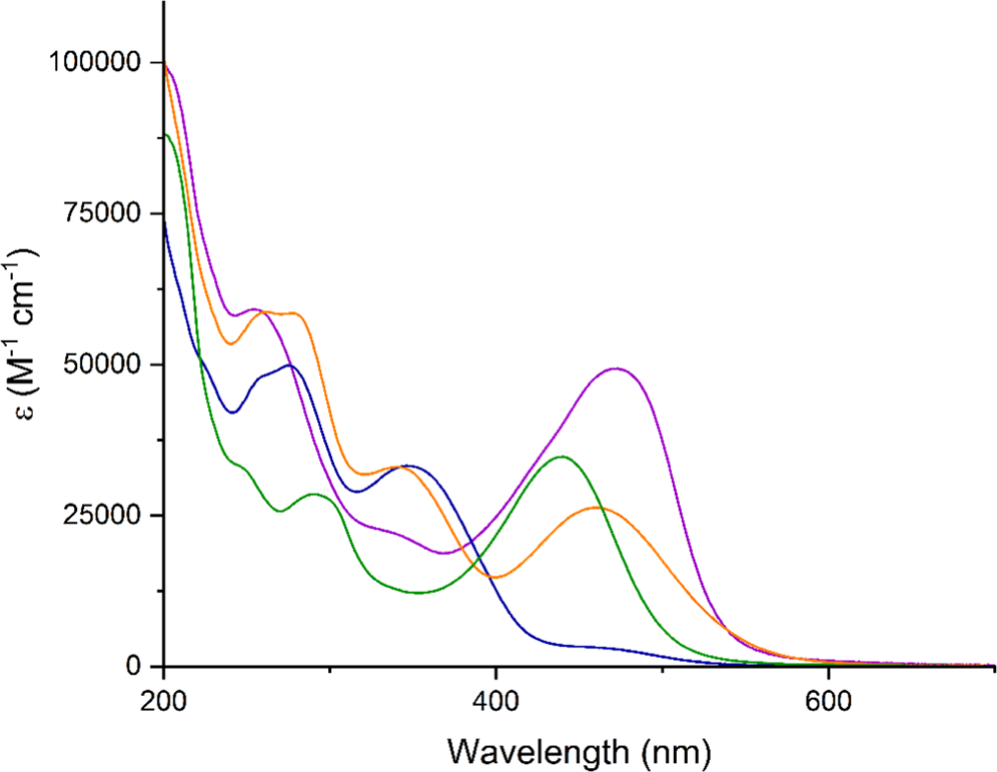
UV–vis spectra of [NBu_4_]_4_[**1**] (purple), [NBu_4_]_4_[**2**] (navy), [NBu_4_]_4_[**3**] (orange), and [NBu_4_]_2_[**4**] (green) measured in acetonitrile at 298 K.

UV–vis absorption spectra of [NBu_4_]_4_[**1**] to [NBu_4_]_2_[**4**] all reveal Mo–O and π–π* peaks in the 200–300 nm region in addition to lower energy peaks relating to ligand-to-POM charge transfers (LPCT) (Figure 3, Table 2). The 2D anions [**1**]^4–^ to [**3**]^4–^ all show a significant red shift (up to 33 nm) of the LPCT peaks with respect to the dipolar compound [**4**]^2–^. This lowered energy of the LPCT peak resulting from the addition of the second POM electron acceptor suggests the possibility for increased β values, which have an inversely squared relationship with transition energies. Notably, the *meta* donor–acceptor relationship in [**2**]^4–^ results in a much weaker LPCT peak due to weakened electronic communication, with the extinction coefficient showing a nearly 95% reduction compared to that of compound [**3**]^4–^. With regard to the absorption profile, a variety of behaviors is seen in 2D chromophores compared to 1D analogues,^6a,32^ and similar red shifts have been observed in systems where communication between the two acceptors across the π-system lowers the energy of the LUMO/LUMO+x acceptor orbitals.

**Table 2:** UV–Visible Absorption and Electrochemical Data for [NBu_4_]_4_[**1**] to [NBu_4_]_2_[**4**] in Acetonitrile at 298 K

				*E*_1/2_/V vs Fc^0/+^ (Δ*E_p_/*mV)^a^	
Compound	λ_max_^/^nm^b^ (*ε/*10^3^ M^–1^ cm^–1^)	*E*_max_/eV	Assignment	NAr_3_ oxidation	POM reduction
[NBu_4_]_4_[**1**]	255 (59.2)	4.86	O→Mo/π→π*	0.458 (85)	–1.023 (93)
	337 (22.2)	3.68	O→Mo/π→π*		
	473 (49.4)	2.62	LPCT		
[NBu_4_]_4_[**2**]	257 (47.7)	4.82	O→Mo/π→π*	0.568 (93)	–0.966 (117)^c^
	275 (49.9)	4.51	O→Mo/π→π*		
	345 (33.2)	3.59	O→Mo/π→π*		
	469 (3.14)	2.64	LPCT		
[NBu_4_]_4_[**3**]	257 (58.5)	4.82	O→Mo/π→π*	0.455 (61)	–1.005 (146)^c^
	262 (33.1)	4.73	O→Mo/π→π*		
	340 (58.7)	3.65	O→Mo/π→π*		
	461 (26.4)	2.69	LPCT		
[NBu_4_]_2_[**4**]	201 (88.0)	6.17	O→Mo/π→π*	0.415 (91)	–1.070 (77)
	291 (28.5)	4.26	O→Mo/π→π*		
	440 (34.7)	2.82	LPCT		

a Concentrations ca. 10^–5^ M in MeCN.

b Solutions ca. 10^–3^ M in analyte and 0.1 M in [NBu_4_][BF_4_] at a glassy carbon working electrode, scan rate 100 mV s^–1^.

c The wave consists of two closely spaced one electron reductions, as shown by differential pulse voltammetry.

Cyclic voltammetry of the four POM derivatives revealed pseudoreversible [{Mo_6_O_18_NAr}]^2–/3–^ waves in all four compounds, along with destructive [{Mo_6_O_18_NAr}]^3–/4–^ processes at more negative potentials (Table 2, Figure S12). In the *bis*-POM derivatives [**1**]^4–^, [**2**]^4–^, and [**3**]^4–^, these processes are observed as two electron waves with nonideal peak separations (i.e., [**X**]^4–/6–^), suggesting the presence of two closely spaced single-electron transfers and weak communication between the two POM units (*vide infra*). Amine oxidation peaks, also with pseudoreversible behavior, were revealed at ca. 0.45 V vs Fc^0/+^ for [**1**]^4–^ and [**3**]^4–^ and 0.415 V for [**4**]^2–^, showing the electron-withdrawing effect of the additional POM on the donor oxidation; logically, the POM reduction for [**4**]^2–^ is also at more negative potential than those of the *bis*-POM species.

Counterintuitively, a substantial (>100 mV) positive shift is seen for the oxidation peak in [**2**]^4–^, even though the *meta* arrangement of the two POM substituents should produce weaker communication with the donor. The reason for this is unclear, but a comparison of [**2**]^4–^ with [**3**]^4–^ and [**1**]^4–^ is not straightforward. In [**1**]^4–^, the second POM is attached to another phenyl group, instead of the same one, while in [**3**]^4–^, steric clash with the *ortho* POM forces the −NTol_2_ donor to twist out of the plane of the bridge and distorts the N from trigonal planar geometry (*vide supra*). This likely weakens the electron withdrawing effect of the 2,4 POM substitution pattern, while the proximity of the negatively charged *ortho* POM to the −NTol_2_ group may stabilize positive charge, leading to a less positive oxidation potential than expected for [**3**]^4–^. Stabilizing influence and restriction of conformation change by the *ortho*-POM may also explain the smaller peak separation observed for the amine oxidation peak of this species.

Nonideal peak separations in the *bis*-POM compounds [**1**]^4–^, [**2**]^4–^, and [**3**]^4–^ (93, 117, and 146 mV, respectively) suggested nonequivalent reductions of the two POM cores, so we used differential pulsed voltammetry (DPV) to resolve the contributing processes (Table 3 and Figure S13). This found distinct processes for [**2**]^4–^ and [**3**]^4–^, with, respectively, 71 and 86 mV differences (Δ*E*) between the [**X**]^4–/5–^ and [**X**]^5–/6–^ reduction potentials. For [**1**]^4–^, DPV showed only one process, as the two POM cores are chemically equivalent and separated by a greater distance (including donor N). Resolving the processes reveals that the first reduction of [**2**]^4–^ is 75 mV less negative than that of [**1**]^4–^ and 17 mV less negative than that of [**3**]^4–^. This echoes the positive shift in its donor oxidation potentials and can be ascribed to weaker communication with the donor (due to the 1,3,5 geometry), leading to lower electron density on the POMs. To quantify the electronic communication between the two POM cores, comproportionation constants (*K*_com_) were calculated from Δ*E* (*K*_com_ = e^(Δ*E*·*F*/*RT*)^ for a one-electron process) for [**1**]^4–^and [**2**]^4–^ (Table 3).^33^ The values obtained indicate Robin-Day Class I—i.e., electronically isolated—mixed valence behavior for [**1**]^4–^, consistent with the separation of the POMs by a greater distance, including the donor atom, but Class II (weakly communicating) behavior for [**2**]^4–^. This is consistent with results obtained for other phenyl bridged, *bis*-POM systems.^10^ For [**3**]^4–^, the chemically inequivalent POMs mean that calculation of *K*_com_ is not appropriate, but as the same *meta* relationship of the two POM cores is present, it can be assumed a very similar weak coupling of the two redox centers is present, with the increased Δ*E* vs [**2**]^4–^ accounted for by the chemical inequivalence.

**Table 3:** Deconvoluted Electrochemical Processes and *K*_com_ Values of [NBu_4_]_4_[**1**] to [NBu_4_]_4_[**3**]

Compound	*E*_1_/V^a^	*E*_2_/V^a^	Δ*E*_(1–2)_^b^/mV	*K*_com_
[NBu_4_]_4_**[1**]	–1.067	–1.067	0	1
[NBu_4_]_4_**[2**]	–0.992	–1.062	70	15
[NBu_4_]_4_**[3**]	–1.009	–1.096	87	–

a *E*_pc_ of reductive process vs Fc^0/+^. *E*_1/2_ will be *ca*. 30 mV less negative.

b Obtained by deconvolution of differential pulsed voltammograms.

### Hyper-Rayleigh Scattering

The nonlinear optical responses of all four compounds were evaluated by hyper-Rayleigh scattering (Table 4), using a 1064 nm source for [NBu_4_]_4_[**1**] to [NBu_4_]_4_[**3**]. Due to two photon fluorescence, [NBu_4_]_2_[**4**] had to be measured at 1200 nm; this and the range of geometries necessitate the use of orientationally averaged static hyperpolarizabilities β_0_,_HRS_ for meaningful comparison between the different chromophores (rather than β_0_,_*zzz*_ used for dipolar systems in our previous work).^2^ Notably, [NBu_4_]_4_[**2**] gives a much smaller β_0,HRS_ than seen for any other compound measured in this work and lower than obtained for any other donor functionalized a-POM to date, a likely result of the weakened donor–acceptor coupling due to the *meta* relationship between the donor and acceptors. Of the other *bis*-POM systems, [NBu_4_]_4_[**1**] gave the higher β_HRS_, possibly due to the situation of the POMs on different rings of the triarylamine, resulting in a larger change in dipole moment between the ground and excited states. Charge transfer to the *ortho* POM of [**3**]^4–^ is almost directionally opposed to the *para* POM, and this seems to have a more important effect than the lowered energy of the LPCT. Dipolar compound [NBu_4_]_4_[**4**] almost equals the β_0,HRS_ of **1**, and conversion to β_0,zzz_ gives 175 × 10^–30^ esu (assuming perfectly linear, dipolar behavior), effectively equaling the performance of its diphenylamino analogue,^2d^ the highest performing phenyl-bridged “POMophore” reported so far.

**Table 4:** Experimental Values of Hyperpolarizability, β, for [NBu_4_]_4_[**1**] to [NBu_4_]_2_[**4**] Measured by Hyper-Rayleigh Scattering in Acetonitrile

compound	LPCT λ_max_/nm	β_1064,HRS_^a^/10^–30^ esu	β_1200,HRS_^a^/10^–30^ esu	β_0,HRS_^b^/10^–30^esu	ρ	β_0,zzz_^c^/10^–30^ esu	β_0,zyy_^c^_/_10^–30^ esu
[NBu_4_]_4_**[1**]	473	451	n.d.	75.8	2.64	191	–57
[NBu_4_]_4_**[2**]	469	91	n.d.	16.3	n.d.	n.d.	n.d.
[NBu_4_]_4_**[3**]	461	295	n.d.	59.7	3.89	152^d^	–18^d^
[NBu_4_]_2_**[4**]	440	n.d.	437	72	4.04	180^d^	–19^d^

a Total molecular HRS response without any assumption of symmetry or contributing tensor elements, measured using 1064 or 1200 nm fundamental laser beams. The quoted units (esu) can be converted into SI units (C^3^ m^3^ J^–2^) by dividing by a factor of 2.693 × 10^20^. Experimental errors on HRS and depolarization measurements are ca. 15%.

b Nonresonant, static β estimated from β_HRS_ using the two-state model.^34^

c Static β tensor components derived from the HRS intensity and depolarization ratio using eqs 1–3.

d May be treated by assuming a single dominant tensor component, yielding β_zzz,0_ = 145 × 10^–30^ esu for **3** and 175 × 10^–30^ esu for **4**, with all other components zero.

Due to their nonlinear molecular shapes, the β responses of [NBu_4_]_4_[**1**] to [NBu_4_]_4_[**3**] are expected to show 2D character. Thus, we attempted to measure depolarization ratios (ρ) for all four compounds, with [NBu_4_]_4_[**4**] providing a direct, linear push–pull comparison for the 2D systems. The ideal value of ρ for a linear push–pull chromophore is 5, and like many other linear push–pull chromophores, the measurement on [**4**]^2–^ produces a lower value, but the drop between this (4.04) and that obtained for *C*_2v_ [**1**]^4–^ (2.64) is substantial, indicating a strongly 2D response in [**1**]^4–^. Due to weak signal, we were unable to obtain ρ for the other *C*_2v_ system [**2**]^4–^; however, [**3**]^4–^ provides an interesting counterpoint. Here, the 2,4 relationship of POMs to the donor on the phenyl bridge results in only a slight lowering of ρ compared to [**4**]^2–^, indicating that the additional POM produces a distorted/dysfunctional 1D dipole rather than a truly 2D system. Extraction of tensor components β_0,zzz_ and β_0,zyy_ results in the estimation of a strongly 2D, off-diagonal response for [**1**]^4–^. For comparison with computed values, tensor components have also been extracted for [**3**]^4–^ and [**4**]^2–^, although their symmetries and depolarization ratios suit treatment as 1D dipoles, yielding β_0,zzz_ = 145 × 10^–30^ and 175 × 10^–30^ esu, respectively

### DFT and TD-DFT Calculations

Structures of the anions [**1**]^4–^ to [**4**]**^2–^** were optimized in Gaussian 2016 at the ωB97X-D, 6-311G(d)/LanL2TZ, IEF-PCM (acetonitrile) level of theory, and linear (UV–vis absorption) and nonlinear optical properties calculated by TD-DFT at the same level of theory. The computed geometries (Table S3) generally show a good match to the experimental structures of the POM derivatives: it is worth noting that in [**2**]^4–^, the significant bending (151.9°) in the C–N–Mo angle to one of the POMs found computationally is observed in the X-ray structure (158.4°), although its origin is not clear.

The TD-DFT computed vertical transition energies and oscillator strengths (Table 5, Figure S14) are consistent with experimental UV–vis spectra in showing a red shift between linear, ditolylamino-donor [**4**]^2–^ and *C*_*2v*_ monotolylamino *bis*-polyoxometalate [**1**]^4–^. Both of these anions have aryl bridges substituted with only one polyoxometalate. The calculations also reproduce the red-shift between [**3**]^4–^ and [**2**]^4–^, the anions where an aryl bridge is substituted with two POMs, although they do not reproduce the experimentally observed red-shift between mono-POM [**4**]^2–^ and [**3**]^4–^ and [**2**]^4–^. This is most pronounced for [**3**]^4–^, where the main band is in fact blue-shifted vs [**4**]^2–^ in the computed spectra, although a weaker, lower energy band is also computed at 2.69 eV (461 nm). Here, the close proximity of the tolyl group and *ortho*-POM in the [**3**]^4–^ structure is the likely explanation, as restricted rotation of the tolyl group will change the distribution of solution conformations that contribute to the experimental spectrum. Moreover, distorted structures can lead to the formation of different CT excited states and thus different transition energies,^35^ and interactions between bulky substituents influence ground-to-excited state geometry changes and thus vibrational effects, which are not taken into account by TD-DFT vertical transition energies.^36^ In addition, through-bond and through-space electronic interactions, which will be present between tolyl and *ortho*-POM in [**3**]^4–^, may be differentially affected by the PCM solvation model.

**Table 5:** TD-DFT Computed Electronic Transitions, 1064 nm, 1200 nm and Static β Responses, and Static Tensor Components *β*_0,zzz_ and *β*_0,zyy_^a^

	LPCT λ_max_^b^	LPCT *E*_max_ (f)^b^/ eV	β_1064,HRS_/c10^–30^ esu	β_1200,HRS_/c10^–30^ esu	β_0,HRS_/d10^–30^ esu	β_0,zzz_/e10^–30^ esu	β_0,zyy_/e10^–30^ esu
[NBu_4_]_4_**[1**]	409	3.03 (2.08)	141.7	105.4	69.6	175.0	–53.1
[NBu_4_]_4_**[2**]	390	3.18 (0.15)	16.4	13.8	13.0	33.3	–4.8
[NBu_4_]_4_**[3**]	376	3.30 (0.98)	105.0	74.7	49.2	124.4	–15.5
[NBu_4_]_2_**[4**]	390	3.18 (1.42)	124.4	94.2	67.0	165.0	–8.6

a All calculations were carried out by TD-DFT at the ωB97X-D/6-311G(d)/LanL2TZ level of theory with acetonitrile solvation by IEFPCM.

b Vertical transitions.

c Dynamic HRS β responses, calculated at 1064 and 1200 nm.

d Static responses calculated at λ = ∞ nm.

e Effective static tensor components deduced from computed ρ and β_0,HRS_ values, assuming planar C*_2v_* symmetry.

**Figure 4:**
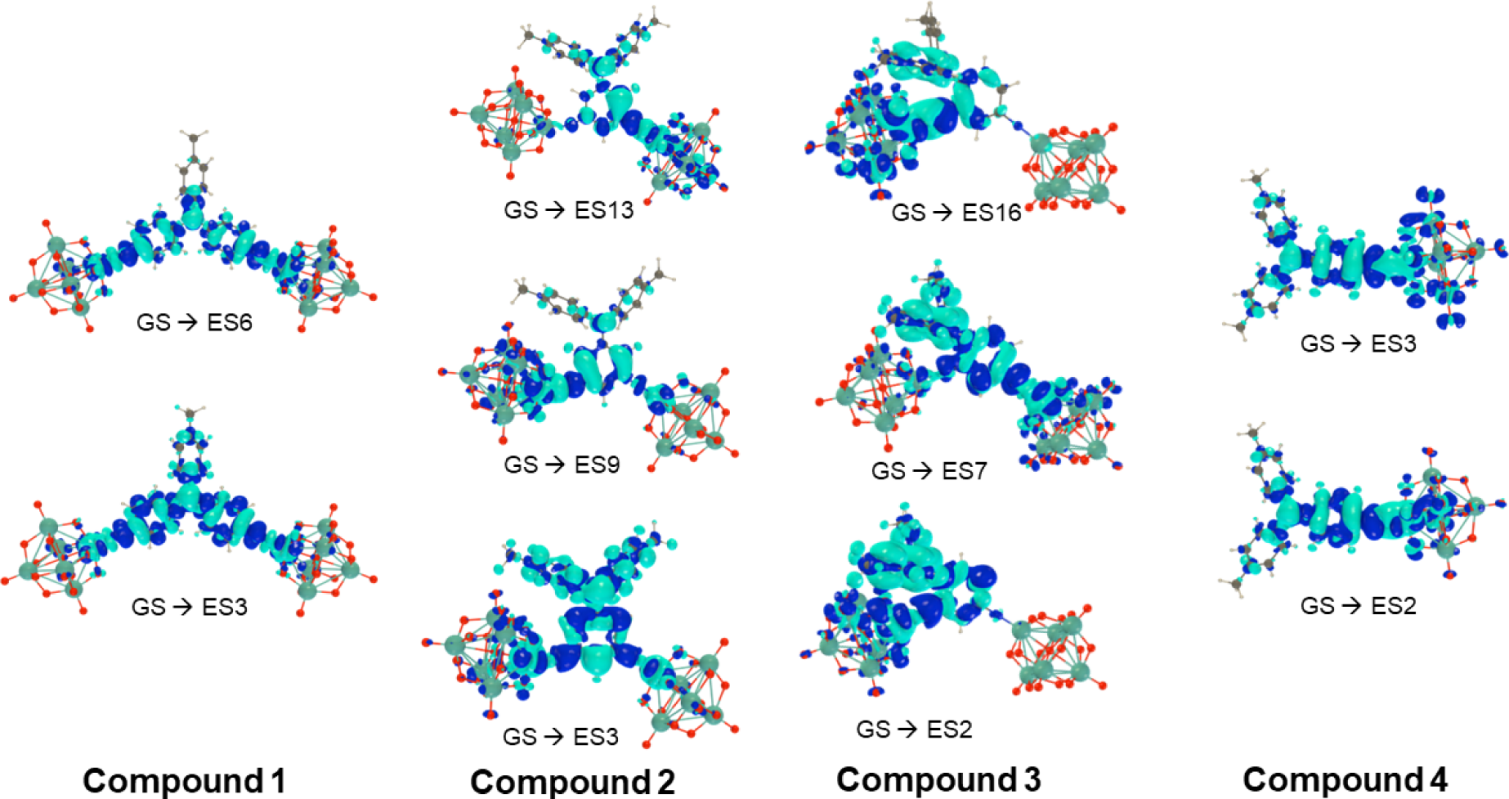
Change of electron density Δ*ρ = ρ_e_– ρ*_*g*_ between the ground and dominant low-energy excited states for [**1**]^4–^ to [**4**]^2–^ calculated at the IEFPCM (solvent = acetonitrile) TDDFT/ωB97X-D/6-311G(d)/LanL2TZ level of approximation (isovalue = 0.0008 au). Light/dark blue corresponds to negative/positive Δρ so that the excitation-induced electron transfer goes from light to dark blue.

**Figure 5:**
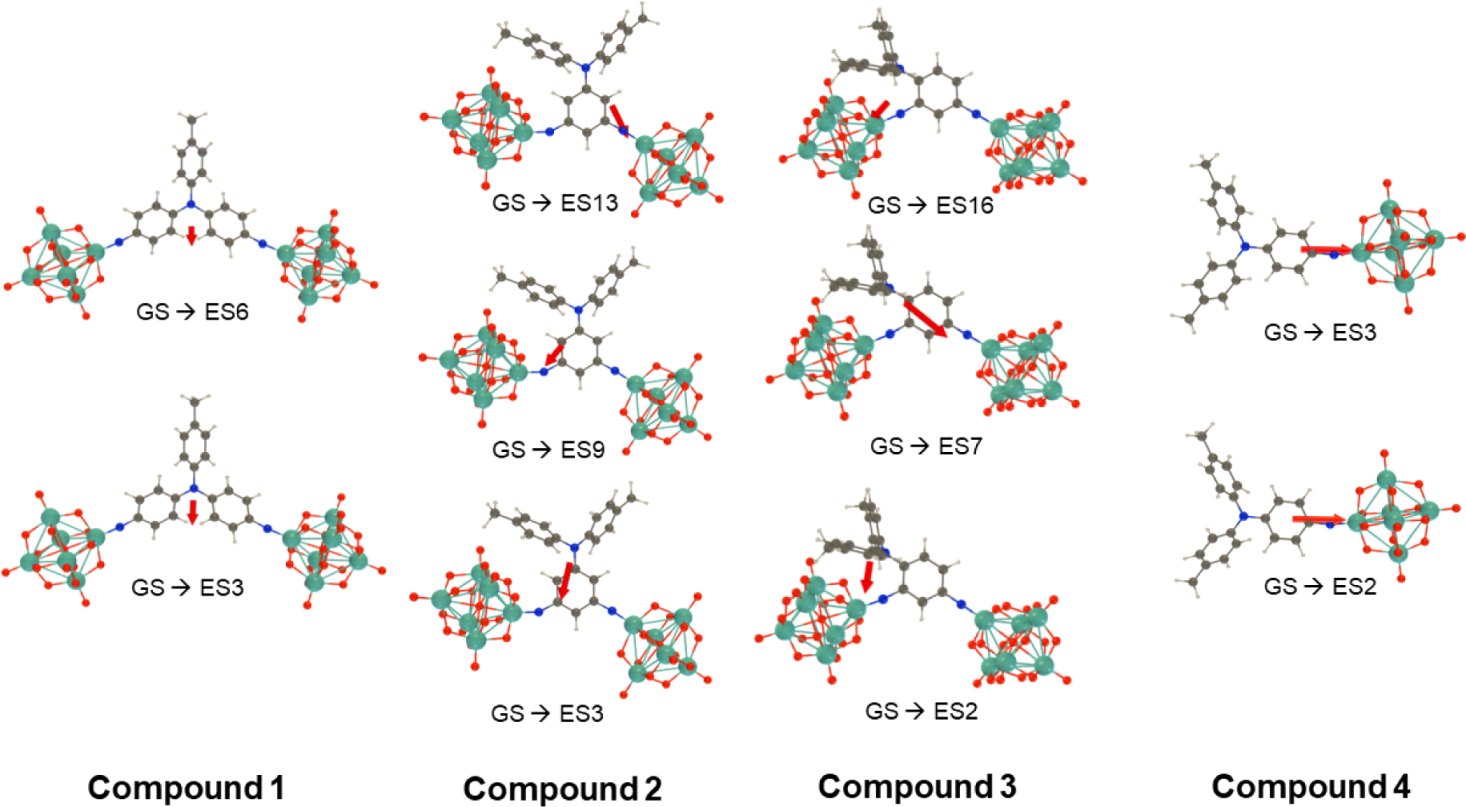
Charge transfer vector (from the negative to the positive barycenter of Δρ) upon excitation from GS to the *n*th ES as evaluated at the IEF-PCM (acetonitrile)/TDDFT/ωB97X-D/6-311G(d)/LanL2TZ level. The nonequilibrium solvation approach was adopted.

Computed changes in the electron density distribution (Δρ) for the dominant transitions are graphically represented in Figure 4, and computed charge transfer vectors are represented in Figure 5. Generally, a shift in electron density is seen from the donor groups to the POM and imido-N, consistent with the expected charge transfer nature of the transitions. Comparing [**1**]^4–^ with one-dimensional analogue [**4**]^2–^, it can be seen that [**4**]^2–^ shows similar behavior to other aryl-bridged one-dimensional POMophores,^2,4,26^ with significant charge transfer onto the POM. However, [**1**]^4–^ shows less involvement of the POM orbitals in either excited state. This is similar to the picture in extended 1D POMophores, where larger π-conjugated systems result in the lower energy transitions being located more within the organic system. The *C*_*2v*_ geometry, with a wide (ca. 120°) angle between the two acceptors, results in a far smaller computed dipole moment change *Δμ*_ge_ than for [**4**]^2–^ (Table S4), as the electron density change is far less dipolar. In [**2**]^4–^ and [**3**]^4–^, the single aryl unit results in more involvement of the POM groups in the CT transitions, although in [**2**]^4–^, they are weakened by the *meta* donor/acceptor geometry, and the A–D–A angles are narrower, resulting in larger *Δμ*_ge_ than in [**1**]^4–^. However, compared to [**4**]^2–^, the additional POM and more complex directionality of CT do reduce *Δμ*_ge_, as shown by the various charge transfer vectors contributing to the overall *Δμ*_ge_. (Figure 5).

**Figure 6:**
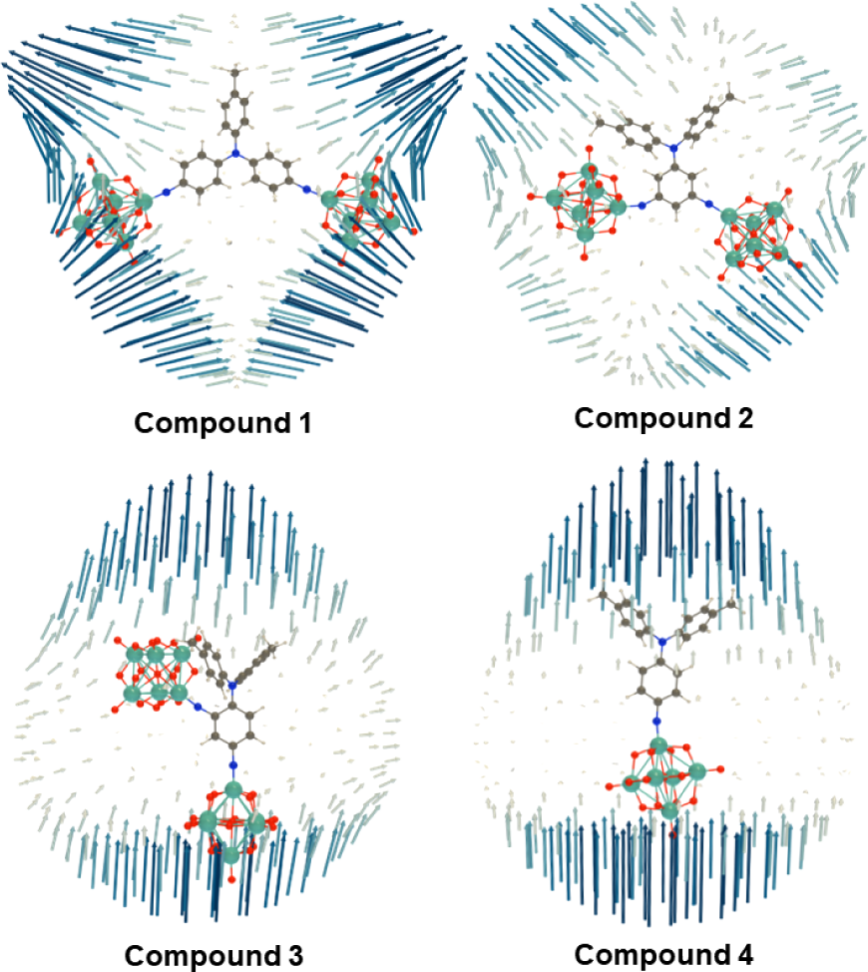
Unit sphere representation (USR) of the first hyperpolarizability tensor (λ = 1064 nm) of **1** to **4** at the IEFPCM (acetonitrile) TDDFT/ωB97X-D/6-311G(d)/LanL2TZ level of approximation. USR factor of 0.0005 Å/*a.u.*_*β*_ for **2** (due to weaker response), 0.0001 Å/*a.u.*_*β*_ for the other compounds.

Computed second-order nonlinear optical coefficients β are shown in Table 5. As observed before, the ωB97X-D computed dynamic β values are somewhat lower than those obtained by experiment, but they reproduce experimental trends. The agreement is much better for the static quantities, showing that most of the differences originate from near-resonance effects and the finite width of the absorption band, which is not accounted for in the calculations. The highest orientationally averaged β_HRS_ values are found for [**1**]^4–^ and [**4**]^2–^ and are quite similar, with static β_0_,_HRS_ showing a very slight advantage for the 2D anion [**1**]^4–^. The next most active is compound [**3**]^4–^, with the *ortho*/*para* positioning of the two POMs relative to the ditolyl donor, and finally, the weak donor–acceptor coupling resulting from *meta* substitution produces a weak response for [**2**]^4–^, ca. 25% of that of [**3**]^4–^ by both experiment and computation. Unit sphere representations (Figure 6) show that for the 1D anion [**4**]^2–^, the β response is dominated by a single tensor component directed along the molecular charge transfer axis. Multidimensional response is most pronounced for [**1**]^4–^ due to its geometry with two charge transfer axes defining a *ca*. 120° angle at the donor atom. Assuming Kleinman and planar symmetry, the two tensor components β_*zzz*_ and β_*zyy*_ have been extracted and are consistent with the experiment in revealing a very substantial 2D response for this compound—β_*zyy*_ is ca. 30% of the magnitude of β_*zzz*_. For [**2**]^4–^ and [**3**]^4–^, the computed magnitudes of β_*zyy*_ are much smaller relative to β_*zzz*_ (ca. 14% and 12%, respectively), consistent with experimental findings. Both computation and experiment thus indicate that the construction of 2D chromophores based on a single donor and two POM acceptors is a viable strategy for engineering off-diagonal β-responses and that this is best achieved with an independent π-bridge for each POM. However, gains from reduced reabsorption may be mitigated by the significantly increased visible light absorption of these 2D species.

## Conclusion

Three new two-dimensional arylimido polyoxometalate charge transfer chromophores, and a related linear one-dimensional system, have been synthesized and thoroughly characterized. Experimental and computed UV–visible absorption spectra indicate a red-shift in ligand-to-polyoxometalate charge transfer bands on moving from 1D to 2D architectures, and electrochemical measurements indicate communication between the POM acceptors (Class II mixed valence behavior) when attached to the same aryl bridge. Both experimentally and computationally determined second-order nonlinear optical coefficients indicate strong responses, with the best performing two compounds in the study showing comparable β values to the most active, previously reported aryl-bridged polyoxometalate donor–acceptor systems. Moreover, by connecting two polyoxometalates, via two bridges to the same donor unit, a strongly two-dimensional response is obtained with a substantial off-diagonal component β_*zyy*_. Connecting both POMs to the same bridge, however, in single donor architectures produces either weak overall or minimally two-dimensional responses. The results thus indicate potential design directions for electrochemically switchable 2D polyoxometalate-based chromophores with strong off-diagonal β responses.

## Data Availability

CIF files for the structures of [NBu_4_]_4_[**2**], [NBu_4_]_4_[**3**], and [NBu_4_]_2_[**2**] are available free of charge from the CCDC (deposition numbers 2379023–2379025) via https://www.ccdc.cam.ac.uk/structures/. In addition to the supplementary data and deposited cif files, data can be obtained by contacting the corresponding author and will be deposited at DOI: 10.17635/Lancaster/researchdata/688

## Supporting Information Available

Full synthetic details for organic precursor compounds, including characterization, X-ray crystallographic details including tabulated bond lengths, cyclic voltammograms and differential pulsed voltammograms, full computational details including calculated bond lengths, UV–vis spectra and charge transfer descriptors23790232379025 (PDF)
Cartesian coordinates of computed geometries (XYZ, XYZ, XYZ, XYZ)

## Supplementary Material

ic4c04179_si_001.pdf

ic4c04179_si_002.xyz

ic4c04179_si_003.xyz

ic4c04179_si_004.xyz

ic4c04179_si_005.xyz
