# Supplementary material for: Synthesis and Optical and Nonlinear Optical Properties of Linear and Two-Dimensional Charge Transfer Chromophores Based on Polyoxometalates
Source: Inorg Chem. 2024 Dec 6;63(51):24250–61. doi: 10.1021/acs.inorgchem.4c04179 (PMC11684011; doi:10.1021/acs.inorgchem.4c04179)
Supplement: Supplementary file 1 — ic4c04179_si_001.pdf [file ic4c04179_si_001.pdf]

Supplementary Information for:

Synthesis, Optical and Non-Linear Optical Properties of Linear and 2-Dimensional Charge Transfer Chromophores based on Polyoxometalates

Bethany R. Hood,[a,b] Yovan de Coene,[c] Claire F. Jones,[b] Ivan Lopez Poves,[b] Noah Deveaux,[d] Nathan R. Halcovitch,[a] Benoît Champagne,*[d] Koen Clays,*[d] and John Fielden.*[a,b]

---

[a] Department of Chemistry, Lancaster University, Lancaster, United Kingdom, LA1 4YB
[b] School of Chemistry, University of East Anglia, Norwich, United Kingdom, NR4 7TJ
[c] Department of Chemistry, University of Leuven, Celestijnenlaan 200D, 3001 Leuven, Belgium
[d] Unit of Theoretical and Structural Physical Chemistry, Namur Institute of Structured Matter, University of Namur, B-5000 Namur, Belgium.

1. ^{1}H-NMR and Mass Spectra of POM derivatives $[NBu_4]_4$[1] to [3] and $[NBu_4]_2$[4]

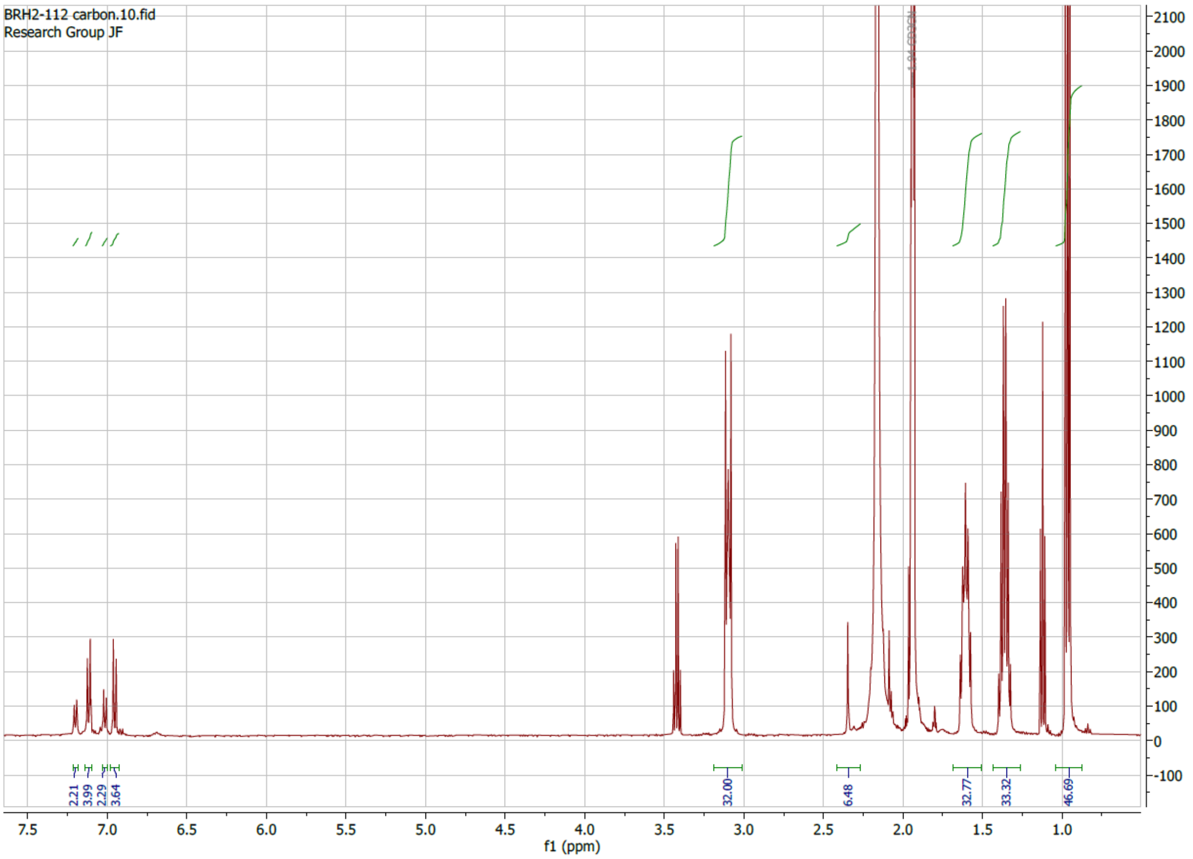

Figure S1 ^{1}H-NMR spectrum of $[NBu_4]_4$[**1**] in CD_3CN.

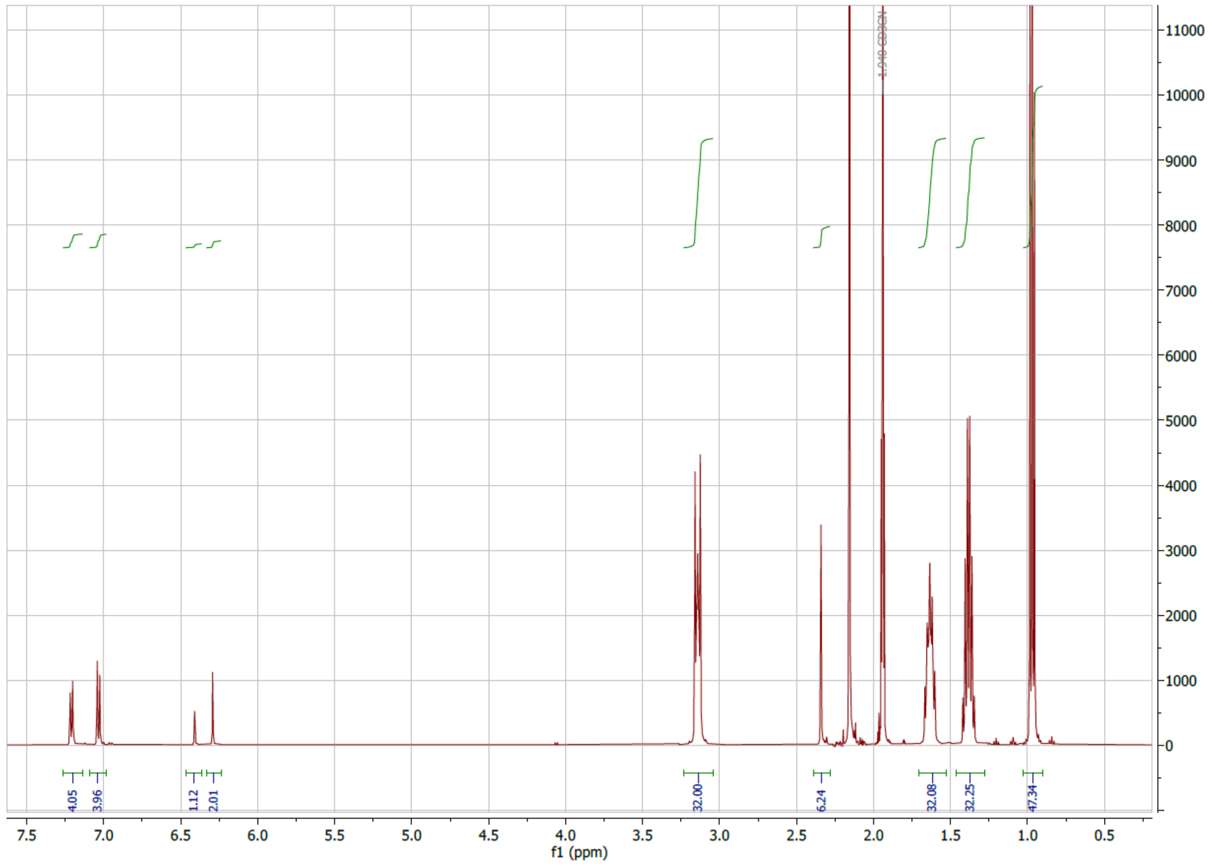

Figure S2 ^{1}H-NMR spectrum of $[NBu_4]_4$[**2**] in CD_3CN.

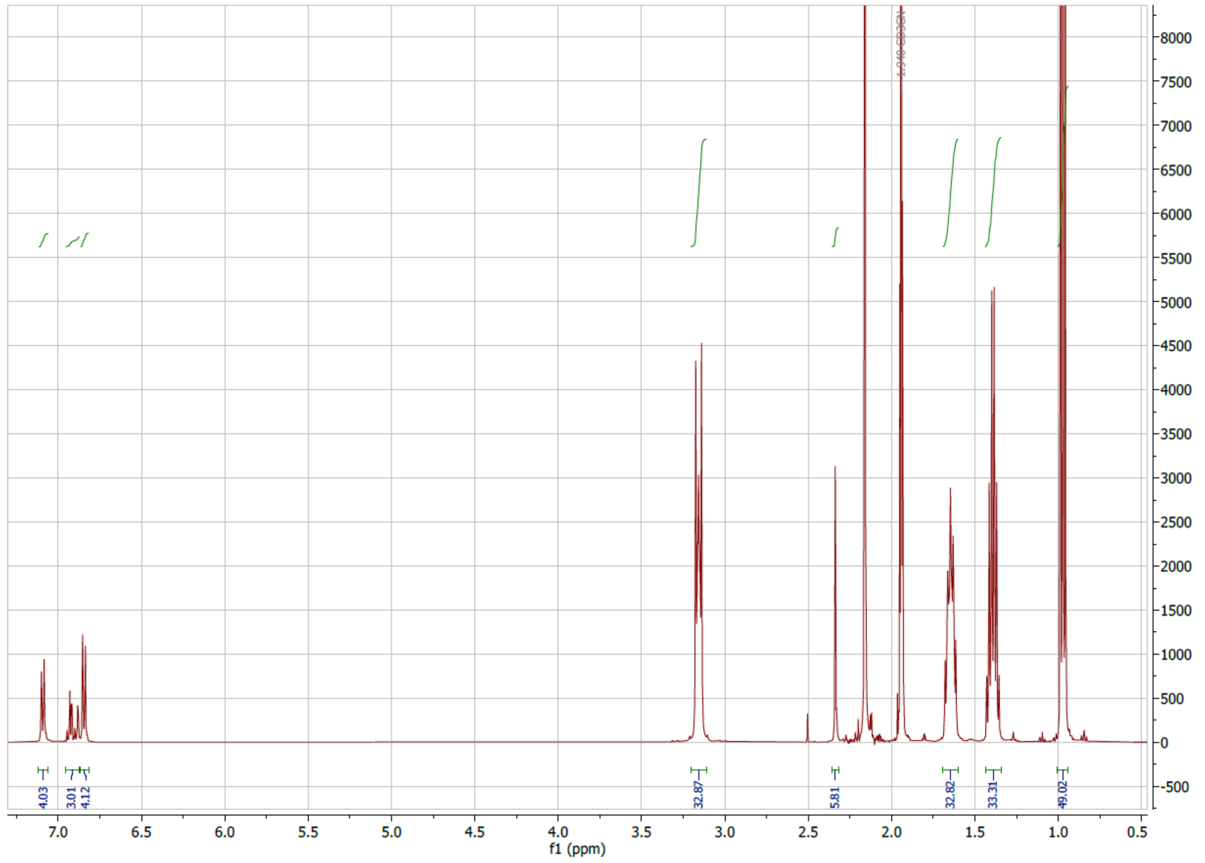

Figure S3 ^{1}H-NMR spectrum of $[NBu_4]_4$[**3**] in CD_3CN.

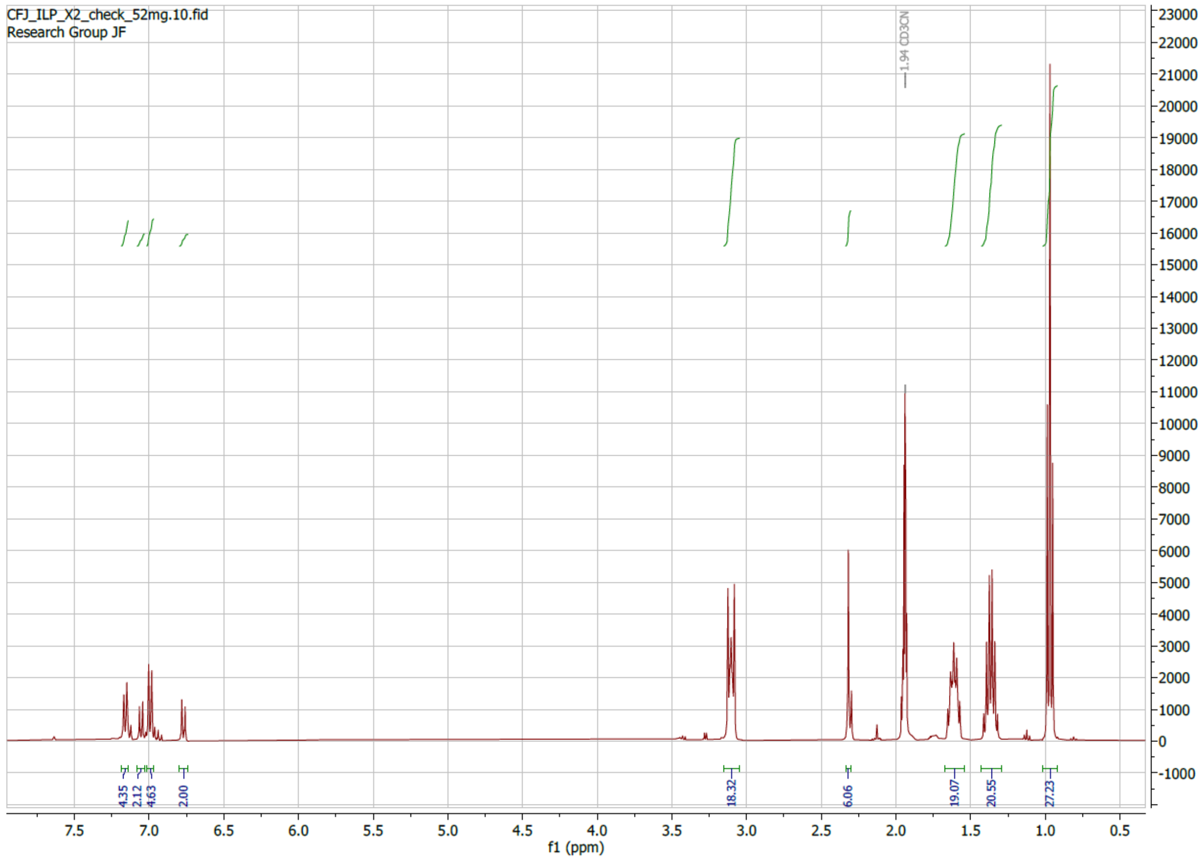

Figure S4 ^{1}H-NMR spectrum of $[NBu_4]_2$[**4**] in CD_3CN.

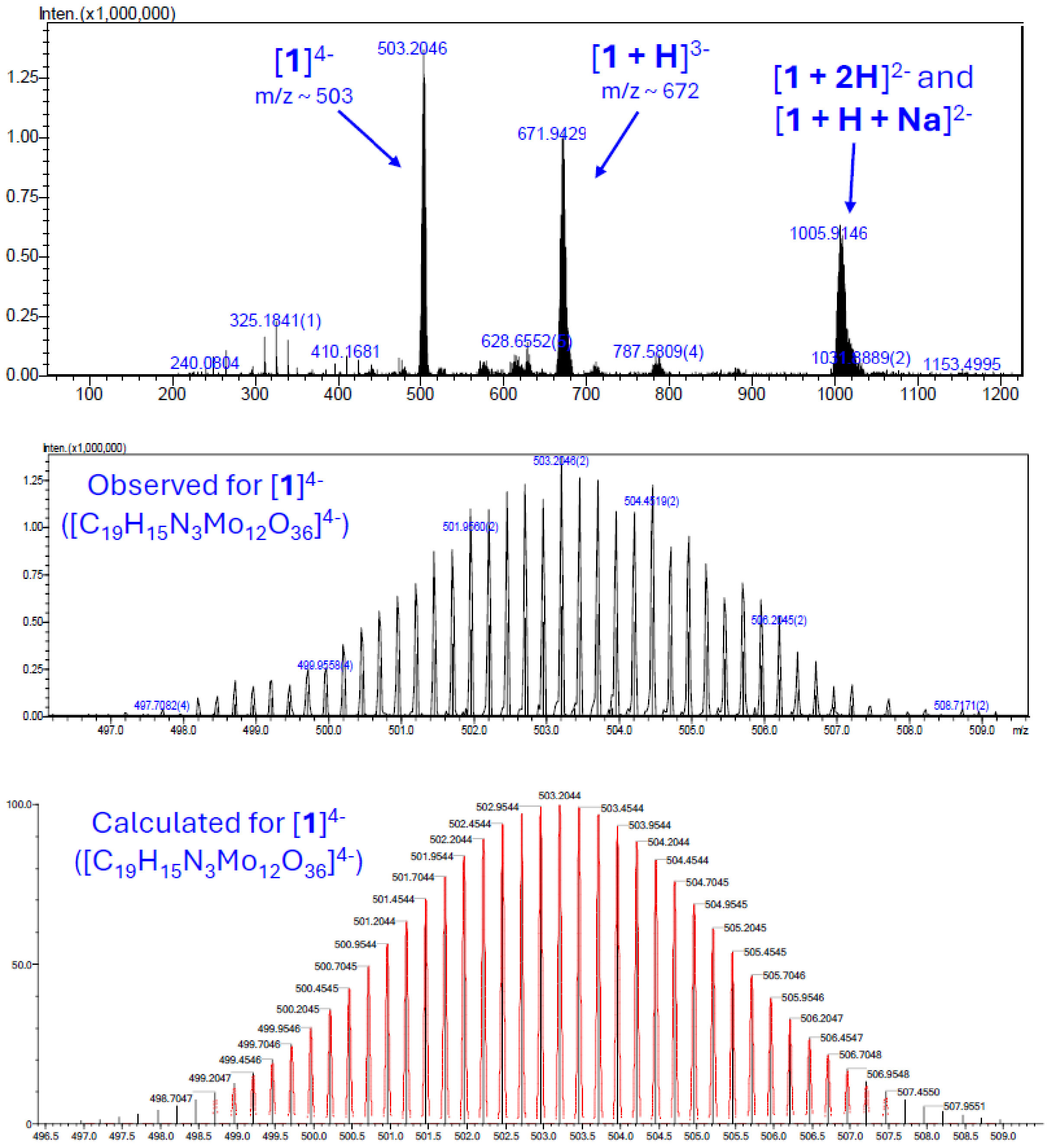

Figure S5 ESI negative mass spectrum of $[NBu_4]_4$[**1**]. Spectrum has been cut for presentation *ca. m/z* = 1200 as there are no significant higher *m/z* signals.

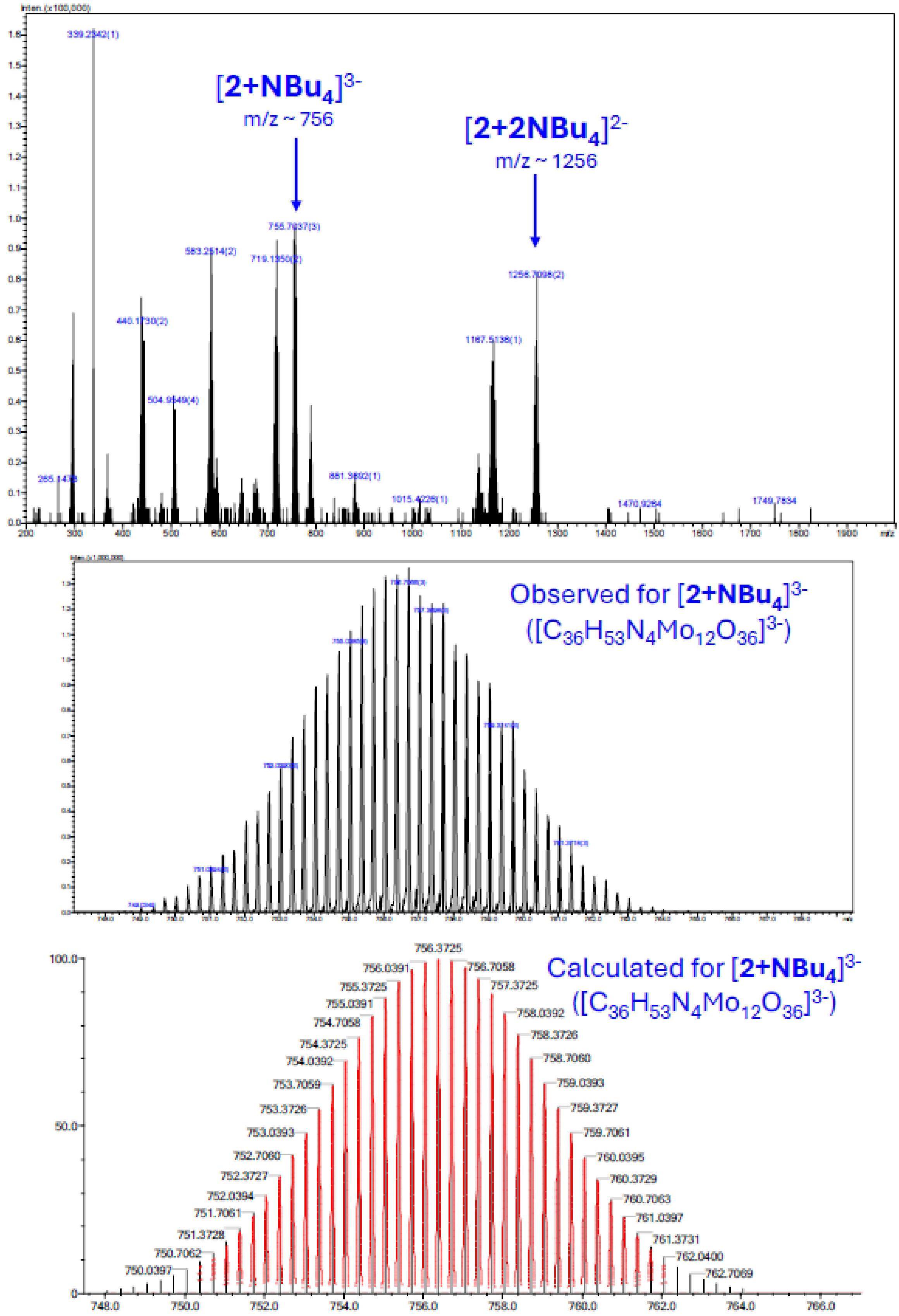

Figure S6 ESI negative mass spectrum of $[NBu_4]_4$[**2**].

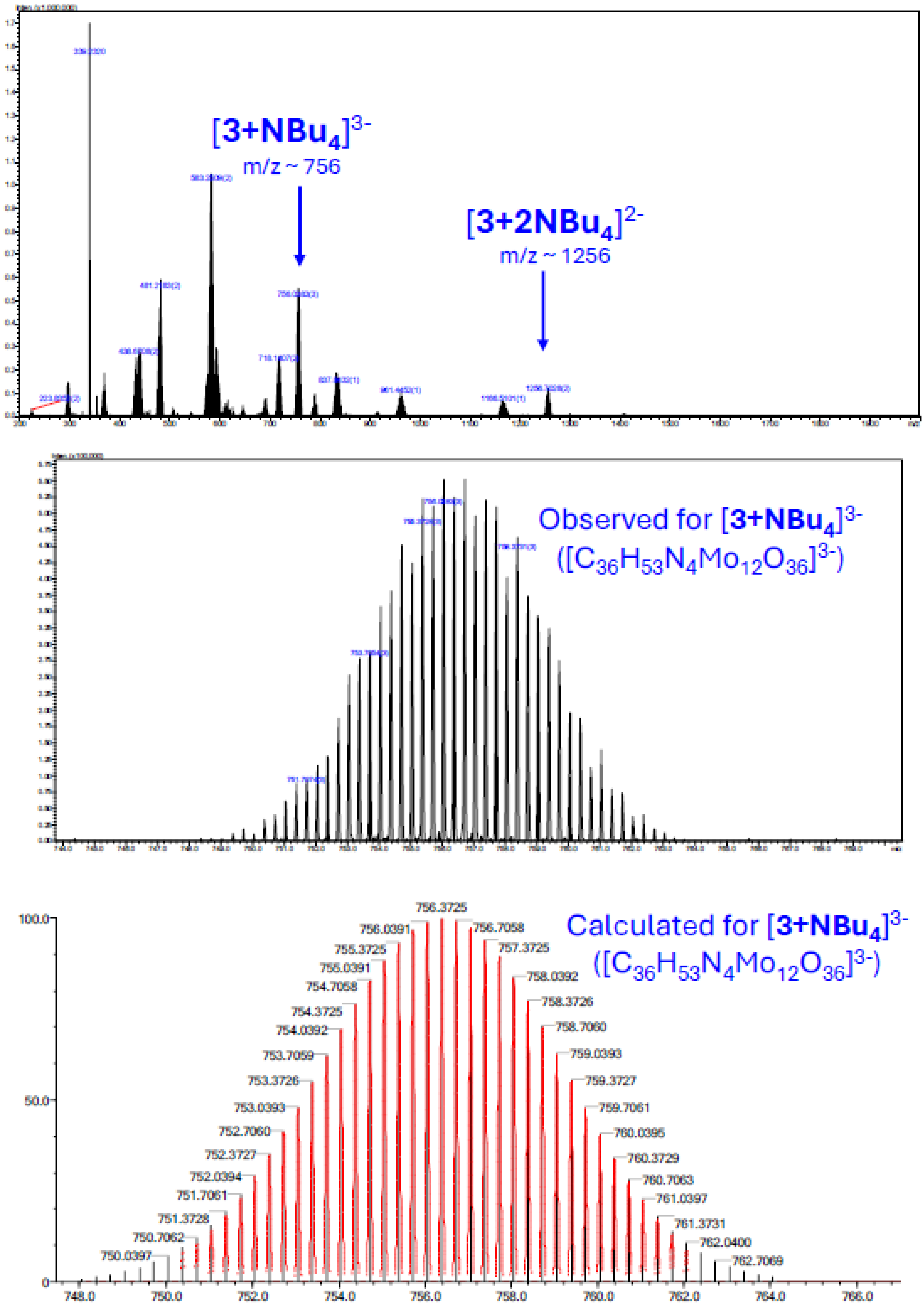

Figure S7 ESI negative mass spectrum of $[NBu_4]_4$[**3**].

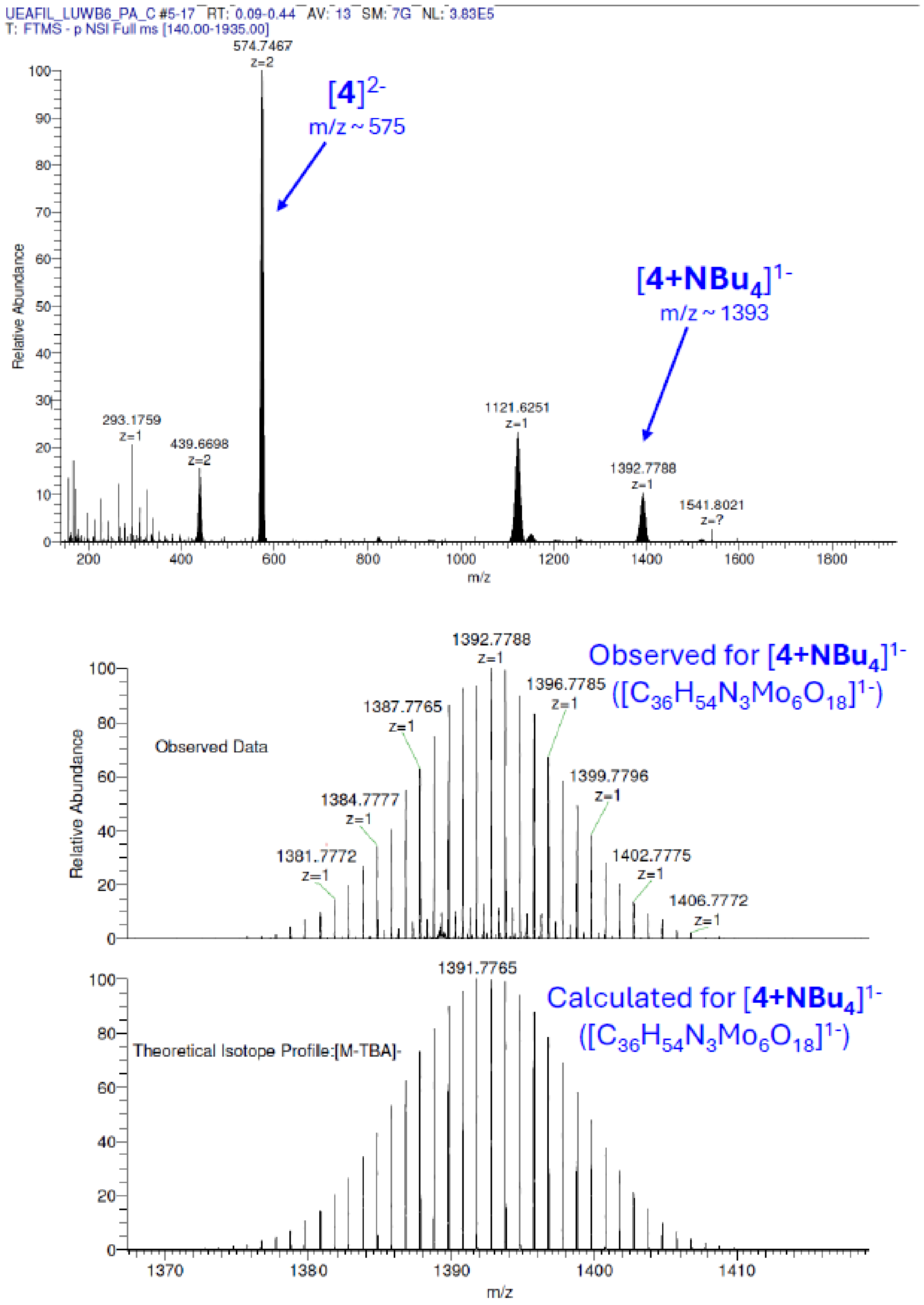

Figure S8 ESI negative mass spectrum of $[NBu_4]_2$[**4**].

2. Additional Synthetic Methods

General Methods and Materials, Synthesis of Polyoxometalate Derivatives. These are reported in the main paper.

Precursor Synthesis. 4,4'-dinitro-4''-methyltriphenylamine (**P1**),[1] 4,4'-diamino-4''-methyltriphenylamine (**P2**),[1] 4-nitro-4',4''-dimethyltriphenylamine (**P7**)[2] and 4-amino-4',4''-dimethyltriphenylamine (**P8**)[2] were synthesised using adaptations of previously reported methods and were characterized only by ^{1}H-NMR as data obtained matched that previously published.[2d] 3,5-dinitro-4',4''-dimethyltriphenylamine (**P3**), 3,5-diamino-4',4''-dimethyltriphenylamine (**P4**), 2,4-dinitro-4',4''-dimethyltriphenylamine (**P5**), 2,4-diamino-4',4''-dimethyltriphenylamine (**P6**) were all prepared by adapting methods used for similar compounds in the literature. The preparation of all organic precursors was carried out under an atmosphere of dry argon using standard Schlenk techniques. The approach to their synthesis outlined overleaf in Scheme S1 and described for each compound below.

Preparation of 4,4'-dinitro-4''-methyltriphenylamine (P1). 1-fluoro-4-nitrobenzene (1.219 g, 8.64 mmol), p-toluidine (0.463, 4.32 mmol), and caesium fluoride (1.500 g, 9.9 mmol) were dissolved in 10 ml of dry dimethylsulfoxide. The resulting yellow solution was heated to 120ºC for 22 hours. The orange precipitate formed on cooling was collected by filtration, washed with water, methanol, then ethanol. The product was purified by purified by recrystallisation from hot DMF/methanol vapour diffusion, to give **P1** as an orange solid (0.512 g, 1.47 mmol) in a 34% yield. ^{1}H-NMR (500 MHz, $CDCl_3$) δ 8.16 – 8.10 (m, 4H), 7.25 – 7.22 (m, 2H), 7.16 – 7.11 (m, 4H), 7.06 (d, J = 8.4 Hz, 2H), 2.40 (s, 3H).

Preparation of 4,4'-diamino-4''-methyltriphenylamine (P2). Compound **P1** (0.294 g, 0.85 mmol) and tin chloride dihydrate (1.25 g, 6.59 mmol) were suspended in 20 ml of dry ethanol and set to reflux for 17 hours. The reaction was poured into ice, made basic with sodium bicarbonate, and then extracted with ethyl acetate. The organic layer was dried over magnesium sulfate, filtered, and then the solvent was removed under vacuum to give compound **P2** a dark orange solid (0.255g, 0.88 mmol) in 100% yield. ^{1}H-NMR (500 MHz, $(CD_3)_2SO$) δ 6.88 (dt, J = 7.3, 0.8 Hz, 2H), 6.73 (d, J = 8.6 Hz, 4H), 6.55 (d, J = 8.5 Hz, 2H), 6.53 – 6.48 (m, 4H), 4.90 (s, 4H), 2.16 (s, 3H).

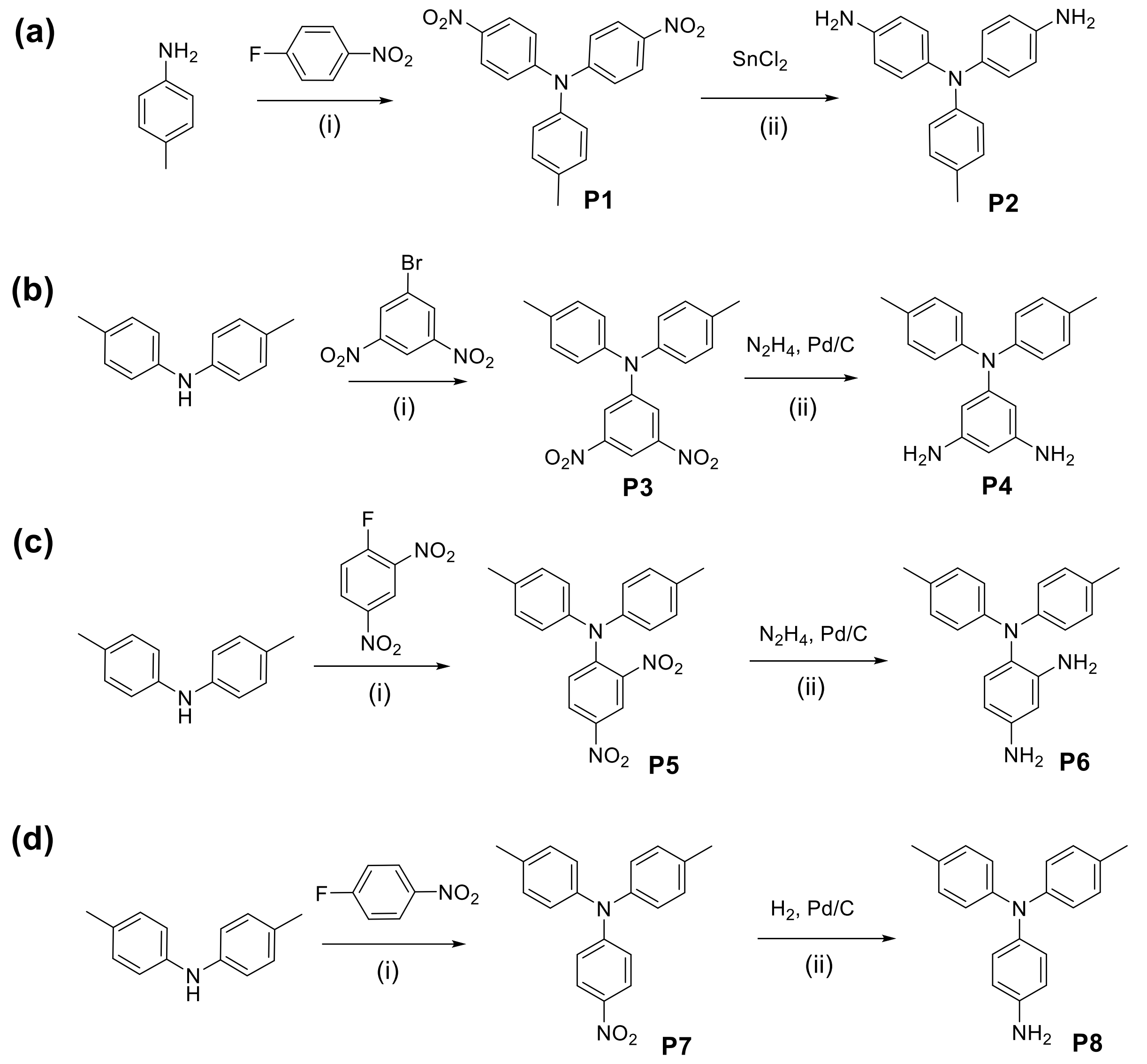

Scheme S1 Synthesis of: (**a**) **P2**, precursor to compound **1**: (i) CsF, DMSO, 120°C, 22 h. (ii) $SnCl_2$, EtOH, reflux, 17 h. (**b**) **P4**, precursor to compound **2**: (i) $PdCl_2(MeCN)_2$, x-Phos, KCO_3, ^tBuOH, reflux, 48 h. (ii) $N_2H_4·H_2O$, 10% Pd/C, EtOH, reflux, 40 h. (**c**) **P6**, precursor to compound **3**: (i) NaH, DMSO, 140°C, 19 h. (ii) $N_2H_4·H_2O$, 10% Pd/C, EtOH, reflux,18 h. (**d**) **P8**, precursor to compound **4**: (i) NaH, DMF, 110°C, 4 h. (ii) H_2, 10% Pd/C, 18 h.

Preparation of 3,5-dinitro-4',4"-dimethyltriphenylamine (P3). 1-bromo-3,5-dinitrobenzene (1.894 g, 11.2 mmol), di-*p*-tolylamine (1.556 g, 7.89 mmol), x-phos (0.329 g, 0.69 mmol), bis(acetonitrile)dichloropalladium(II) (0.060 g, 0.23 mmol) and potassium carbonate (1.483 g, 10.7 mmol) were suspended in 15 ml of dry tert-butanol and the resulting mixture refluxed for between 48 and 112 hours. When cool, the reaction mixture was poured into water, extracted with ethyl acetate, and the organic phase dried with magnesium sulfate, then filtered. The solvent was removed under vacuum to give a dark solid that was purified by washing with hexane and then recrystallising from hot hexane. Compound **P3** was obtained as orange crystals (1.604 g, 4.41 mmol) in a 56% yield. ^{1}H-NMR (500 MHz,$CDCl_3$) δ 8.35 (t, *J* = 2.0 Hz, 1H), 7.94 (d, *J* = 1.9 Hz, 2H), 7.23 – 7.17 (m, 4H), 7.09 – 7.04 (m, 4H), 2.37 (s, 6H). ^{13}C-NMR (126 MHz, $CDCl_3$) δ 150.73, 149.42, 142.49, 136.45, 131.16, 126.11, 117.16,

108.38, 21.17. Anal (Calcd) % for $C_{20}H_{17}N_3O_4$: C, 66.24 (66.11); H, 4.76 (4.72); N, 11.42 (11.56). MS (MALDI) = calcd for $C_{20}H_{17}N_3O_4^+$ 363.12, found 363.05. FTIR: 3103 (vw), 3036 (vw), 2920 (vw), 2863 (vw), 1627 (w), 1609 (vw), 1540 (s), 1508 (s), 1457 (m), 1343 (s), 1327 (sh), 1303 (w), 1280 (m), 1231 (vw), 1213 (vw), 1196 (vw), 1177 (vw), 1112 (w), 1073 (w), 1021 (w), 933 (vw), 908 (w), 875 (w), 848 (w), 832 (w), 814 (m), 725 (s), 696 (sh), 653 (m), 588 (m). UV-vis (MeCN) λ, nm (ε, M^{-1} cm^{-1}): 281 (23.9×10^3); 373 (2.97×10^3).

Preparation of 3,5-diamino-4',4''-dimethyltriphenylamine (P4). Compound **P4** (1.137 g, 3.13 mmol), hydrazine monohydrate (0.6 ml, 12.0 mmol) and palladium on carbon 10% (0.140 g) were suspended in 40 ml of dry ethanol. The mixture was refluxed for 18 hours and then filtered while hot to give a colourless solution which cooled to give pure compound **P4** as colourless crystals (0.625 g, 2.06 mmol) in a 66% yield. ^{1}H-NMR (500 MHz, $(CD_3)_2SO$) δ 7.02 (d, *J* = 8.4 Hz, 4H), 6.83 (d, *J* = 8.4 Hz, 4H), 5.51 (t, *J* = 1.9 Hz, 1H), 5.46 (d, *J* = 1.8 Hz, 2H), 4.65 (s, 4H), 2.22 (s, 6H). ^{13}C-NMR (126 MHz, $(CD_3)_2SO$) δ 149.79, 148.79, 145.53, 130.63, 129.50, 123.51, 99.34, 95.46, 20.32. Anal (Calcd) % for $C_{20}H_{21}N_3$: C, 79.08 (79.17); H, 7.04 (6.98); N, 13.63 (13.85). MS (MALDI) = calcd for $C_{20}H_{21}N_3^+$ 303.17, found 303.23. FTIR: 3392 (m), 3318 (m), 3204 (w), 3020 (vw), 2916 (vw), 2857 (vw), 1592 (s), 1504 (s), 1491 (s), 1474 (sh), 1364 (w), 1317 (w), 1271 (m), 1240 (s), 1182 (s), 1154 (m), 1112 (vw), 1020 (w), 836 (sh), 815 (s), 755 (m), 712 (w), 686 (s), 617 (m), 597 (m), 554 (w). UV-vis (MeCN) λ, nm (ε, M^{-1} cm^{-1}): 253 (18.2×10^3); 300 (21.1×10^3).

Preparation of 2,4-dinitro-4',4''-dimethyltriphenylamine (P5). To a solution of di-4-tolylamine (0.788g, 4.00 mmol) and sodium hydride 60% in oil (0.160) stirred in 4ml dimethylsulfoxide for 20 minutes at room temperature, dinitrofluorobenzene (0.51 ml, 4.03 mmol) was added. After heating at 140°C for 19 hours, the cooled solution was poured into 40 ml water and 40 ml methanol to produce an oily solid collected by filtration. Purification by column chromatography in dichloromethane followed by recrystallisation from hot ethanol gave compound **P5** (0.287 g, 0.790 mmol) as orange crystals in a 20% yield. ^{1}H-NMR (500 MHz, $(CD_3)_2SO$) δ 8.59 (d, *J* = 2.7 Hz, 1H), 8.30 (dd, *J* = 9.3, 2.7 Hz, 1H), 7.20 (d, *J* = 9.3 Hz, 1H), 7.17 (d *J* = 8.5 Hz, 4H), 6.92 (d, *J* = 8.5 Hz, 4H), 2.29 (s, 6H). ^{13}C-NMR (126 MHz, $(CD_3)_2SO$) δ 145.64, 142.80, 140.68, 139.80, 135.21, 130.46, 128.30, 126.31, 124.40, 123.04, 20.48. Anal (Calcd) % for $C_{17}H_{17}N_3O_4$): C, 65.83 (66.11); H, 4.42 (4.72); N, 11.39 (11.56). MS (MALDI) = calcd for $C_{20}H_{17}N_3O_4^+$ 363.12, found 363.11. FTIR: 3101 (vw), 3082 (vw), 2920 (vw), 2862 (vs), 1594 (s), 1533 (w), 1508 (s), 1478 (m), 1421 (vw), 1347 (sh), 1328 (s), 1298 (sh), 1280 (s), 1243 (sh), 1243 (sh), 1214 (m), 1152 (m), 1135 (w), 1113 (w), 1069 (vw), 1044 (vw), 1019 (vw), 978 (vw), 934 (vw), 914 (vw), 898 (m), 833 (vw), 815 (m), 801 (s), 757 (sh), 742 (m), 732 (m), 714 (m), 689 (sh), 652 (w), 623 (vw), 599 (w), 559 (m). UV-vis (MeCN) λ, nm (ε, M^{-1} cm^{-1}): 251 (19.5×10^3); 410 (15.0×10^3).

Preparation of 2,4-diamino-4',4''-dimethyltriphenylamine (P6). To compound **P5** (0.203 g, 0.559 mmol) and Pd/C 10% (0.0237 g) in 8 ml of ethanol, hydrazine hydrate (0.120 ml, g, 2.47 mmol) was added and the resulting mixture heated to reflux for 30 minutes. After the reaction appeared complete by TLC, the solid was removed by filtration and the volume reduced under vacuum to produce an off-white precipitate which was collected by filtration and washed with ethanol to give compound **P6** (0.045 g, 0.148 mmol) as an off-white solid with a 26% yield.

^{1}H-NMR (500 MHz, $(CD_3)_2SO$) δ 7.04 – 6.93 (m, 4H), 6.84 – 6.77 (m, 4H), 6.56 (d, J = 8.2 Hz, 1H), 5.98 (d, J = 2.5 Hz, 1H), 5.86 (dd, J = 8.3, 2.5 Hz, 1H), 4.81 (s, 2H), 4.41 (s, 2H), 2.20 (s, 6H). ^{13}C-NMR (126 MHz, $(CD_3)_2SO$) δ 147.95, 145.7, 144.96, 130.31, 129.37, 129.23, 120.63, 120.02, 104.47, 100.57, 20.22. MS (MALDI) = calcd for $C_{20}H_{21}N_3^+$ 303.17, found 303.28. FTIR: 3483 (w), 3429 (w), 3374 (w), 3338 (w), 3022 (vw), 2917 (vw), 2857 (vw), 1889 (vw), 1620 (m), 1607 (m), 1503 (s), 1460 (w), 1310 (m), 1289 (m), 1270 (sh), 1251 (s), 1206 (w), 1107 (w), 1036 (w), 1020 (vw), 965 (w), 833 (sh), 809 (s), 782 (sh), 755 (vw), 731 (w), 704 (w), 636 (w), 607 (sh), 564 (sh), 547 (m). UV-vis (MeCN) λ, nm (ε, M^{-1} cm^{-1}): 212 (55.0×10^3); 302 (22.9×10^3).

Preparation of 4-nitro-4',4''-dimethyltriphenylamine (P7). NaH (1.22g, 50.69 mmol) was suspended in anhydrous DMF (50 mL) at 0˚C. Ditolylamine (10g, 50.69 mmol) in anhydrous DMF (50 ml, anhydrous) was cooled to 0˚C, and added dropwise to the suspension of NaH before stirring for 1 hour while warming to room temperature. 4-nitro-fluorobenzene (7.15g, 50.69 mmol) was added, and the reaction mixture refluxed at 110°C for 4 hours. After cooling to room temperature water was slowly added (CAUTION – potential for reaction with residual NaH) to the reaction mixture, resulting in production of a precipitate that was recovered by filtration and washed with water. Recrystallisation from hot ethyl acetate (cooled overnight at -18˚C) produced compound **P7** as a flaky orange solid (11.30g, 35.48 mmol, 70%). ^{1}H-NMR (400 MHz, $CDCl_3$) δ: 7.00 (4H, *d*, J_{HH} = 7.0 Hz), 6.94-6.91 (m, 6H), 6.62 (*d*, J_{HH} = 7.0 Hz, 2H), 2.28 (s, 6H).

Preparation of 4-amino-4',4''-dimethytriphenylamine (P8). Compound **P7** (11.30g, 35.48 mmol) was dissolved in a mixture of ethyl acetate and methanol (75 ml:75 ml) and degassed thoroughly by bubbling with Ar. 10% Pd/C (1 g) was added as a solid and the reaction mixture was degassed again with Ar, before an H_2 atmosphere was introduced by balloon and venting. A positive pressure of H_2 was maintained for 18 h using an H_2 filled balloon, with stirring at room temperature. Pd/C was removed by filtration, and the solvent removed *in vacuo* before redissolution in DCM, washing with H_2O and drying over $MgSO_4$. Removal of solvent *in vacuo* and reprecipitation from EtOAc by addition of n-hexane yielded compound **P8** as an off-white solid (5.88g, 20.4 mmol, 57%). ^{1}H-NMR 400 MHz, $CDCl_3$ δ: 8.00 (d, J_{HH} = 7.0 Hz, 2H), 7.17 (*d*, 4H), 7.05 (*d*, J_{HH} = 7.0Hz, 4H), 6.85 (d, 2H), 3.55 (s, 2H), 2.36 (s, 6H).

2. X-ray Crystallographic Details

Table S1. Crystallographic Data and Refinement Details for $[NBu_4]_4$[**2**], $[NBu_4]_4$[**3**] and $[NBu_4]_2$[**4**]

	$[NBu_4]_4$[**2**]•$(CH_3)_2CO$•H_2O	$[NBu_4]_4$[**3**]•H_2O	$[NBu_4]_2$[**4**]•EtOAc
Formula	$C_{87}H_{169}Mo_{12}N_7O_{38}$	$C_{84}H_{163}Mo_{12}N_7O_{37}$	$C_{56}H_{98}Mo_6N_4O_{20}$
M	3072.55	3014.47	1723.02
crystal system	monoclinic	orthorhombic	monoclinic
space group	*I*2/a	*P*bca	*P*2$_1$/c
a/Å	26.7661(3)	31.7241(3)	11.5344(2)
b/Å	23.4821(3)	22.5870(2)	16.1887(2)
c/Å	39.9075(3)	32.6291(4)	36.2118(5)
α/deg	90	90	90
β/deg	92.159(1)	90	91.628(1)
γ/deg	90	90	90
V/Å^3	25065.0(5)	23380.5(4)	6759.0(2)
Z	8	8	4
Radiation	Mo Kα (λ = 0.71073 Å)	Mo Kα (λ = 0.71073 Å)	Cu Kα (λ = 1.54184 Å)
T/K	99.99(14)	100.0(1)	100.0(1)
μ/mm^{-1}	1.228	1.314	9.438
Cryst. size/mm	0.7 × 0.44 × 0.36	0.825 × 0.249 × 0.144	0.825 × 0.249 × 0.144
Cryst. description	clear dark orange block	clear dark orange block	clear dark orange plate
No. reflns collected	337868	557475	48386
No. of indep. reflns (R_{int})	25609 [R_{int} = 0.0500]	21376 [R_{int} = 0.0693]	13479 [R_{int} = 0.0645]
θ_{max}/deg (completeness)	26.372 (100.0%)	25.350 (99.9%)	77.436 (93.8%)
θ_{full}/deg (completeness)	25.242 (100.0%)	25.242 (99.9%)	67.684 (99.5%)
Reflections with $I > 2\sigma(I)$	21503	19596	11268
Goodness-of-fit on F^2	1.092	1.234	1.031
final R_1, wR_2 [$I > 2\sigma(I)$]^a	R1 = 0.0669, wR2 = 0.1833	R1 = 0.0755, wR2 = 0.1846	R1 = 0.0657, wR2 = 0.1760
(all data)	R1 = 0.0784, wR2 = 0.1945	R1 = 0.0811, wR2 = 0.1877	R1 = 0.0765, wR2 = 0.1843
Peak and hole/e Å^{-3}	1.50 and -0.81	1.90/-0.80	2.31/-1.72

Structures and Selected Bond Lengths. Representations of the asymmetric units of $[NBu_4]_4$[**2**]•$(CH_3)_2CO$•H_2O, $[NBu_4]_4$[**3**]•H_2O and $[NBu_4]_2$[**4**]•EtOAc are provided below in Figures S1 to S3.

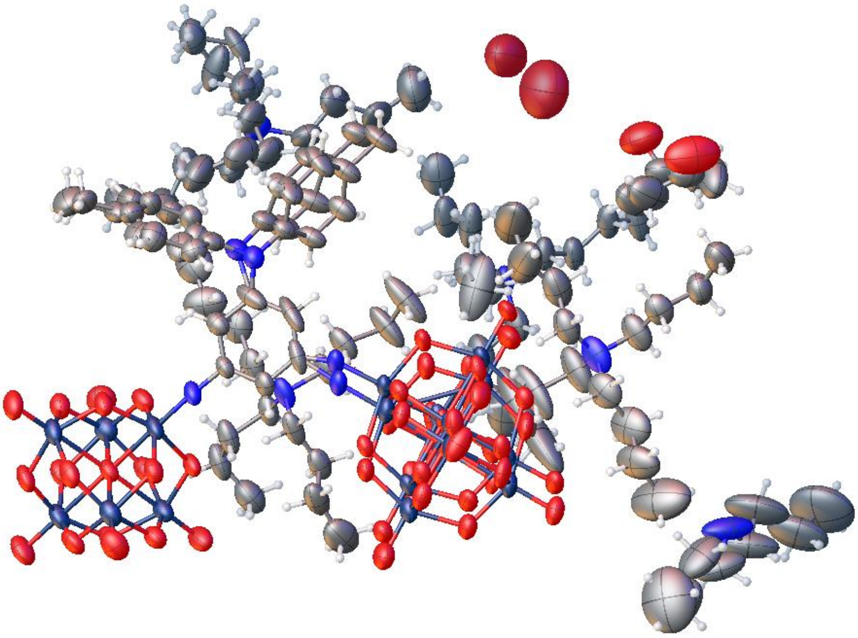

Figure S9. Representation of the asymmetric unit in $[NBu_4]_4$[**2**]•$(CH_3)_2CO$•H_2O. Thermal ellipsoids are at the 30% probability level. The structure shows disorder in the positions of one of the {Mo_6} unit, in the -$NTol_2$ group, one of the NBu_4^+, and the acetone molecule. The isolated O atoms are a water molecule disordered over two positions, one $[NBu_4]^+$ is on a special position (and is fully seen when the structure is grown). Colour scheme: Mo is navy; O, red; C, gray; N, blue; H atoms are represented by white spheres of arbitrary radii.

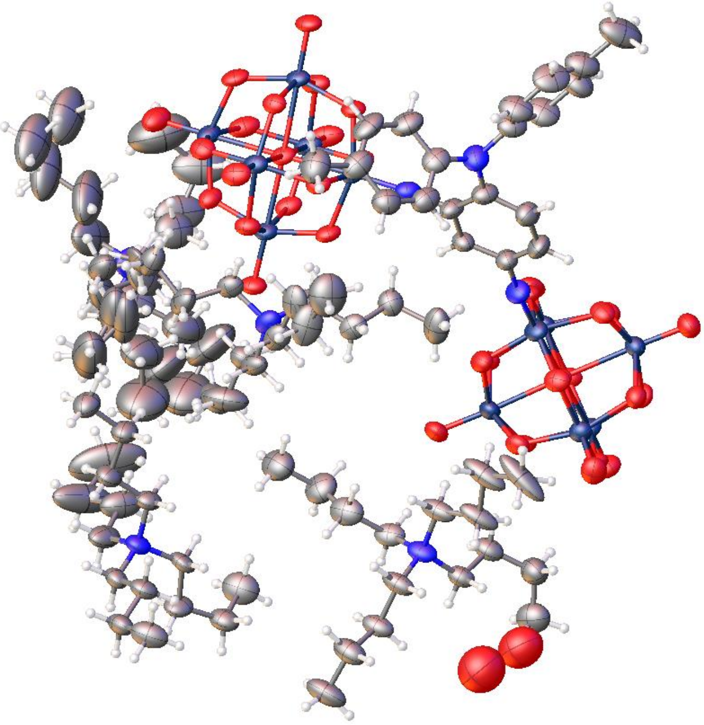

Figure S10. Representation of the asymmetric unit in $[NBu_4]_4$[**3**]•H_2O. Thermal ellipsoids are at the 30% probability level. The structure shows disorder in one of the NBu_4^+, and the isolated O atoms are a water molecule disordered over two positions. Colour scheme: Mo is navy; O, red; C, gray; N, blue; H atoms are represented by white spheres of arbitrary radii.

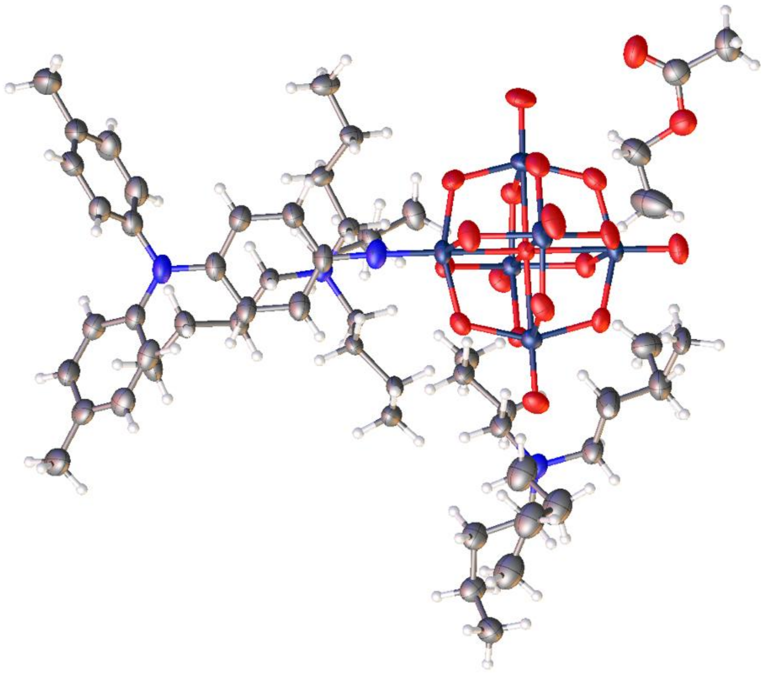

Figure S11. Representation of the asymmetric unit in $[NBu_4]_2$[**4**]. Thermal ellipsoids are at the 30% probability level. The structure shows disorder in one of the NBu_4^+. Colour scheme: Mo is navy; O, red; C, gray; N, blue; H atoms are represented by white spheres of arbitrary radii.

Selected bond lengths and angles for the anions are collected in Table S2. The structures show the typical imido-Lindqvist pattern of a shortened bond length from the imido-Mo (Mo^{im}) to the central oxygen (O^c), lengthened equatorial bond lengths from Mo^{im} to the oxygens bridging to the belt Mo positions (Mo^b), and a lengthened axial bond length from the *trans*-Mo (Mo^t) to O^c.[3] The most remarkable feature of the strictures is the unusually bent Mo-N-C angle in one of the disordered parts of [**2**]$^{4-}$, the reason for this unclear but the presence of a similar (tighter) angle in computed structures (Table S3) implies that it may be due to intermolecular steric/charge-charge repulsion between the two $\{Mo_6\}$ units, rather than simply crystal packing. Like other arylamino donor imido-Lindqvist systems,[2a] there is little or no evidence of quinoidal behavior in the phenyl bridges of any of the structures.

Table S2. Significant bond lengths (Å) and angles (˚) of the {$Mo_6O_{18}N$} clusters of [NBu_4]$_4$[**2**], [NBu_4]$_4$[**3**] and [NBu_4]$_2$[**4**].[a]

	**C-N-Mo^{im}**	**N-Mo^{im}**	**Mo^{im}-O^c**	**Mo^t-O^c**	**Mo^{im}-O^b**	**Mo^t-O^b**
**[2]$^{4-}$** max	172.5(4)	1.751(13)	2.211(8)	2.359(3)	2.005(5)	1.957(12)
min	158.4(14)	1.734(15)	2.180(3)	2.345(9)	1.907(5)	1.861(4)
**mean**	167.2(10)	1.743(11)	2.194(7)	2.350(7)	1.945(8)	1.917(9)
**[3]$^{4-}$** max	175.6(8)	1.738(8)	2.211(6)	2.360(6)	2.020(7)	1.981(7)
min	167.7(7)	1.737(9)	2.208(6)	2.348(6)	1.880(7)	1.883(6)
**mean**	171.7(7)	1.738(9)	2.210(6)	2.354(6)	1.954(7)	1.921(7)
**[4]$^{2-}$**	173.7(6)	1.740(6)	2.205(4)	2.349(4)	max: 1.967(4)<br>min: 1.936(5)<br>mean: 1.948(5)	max: 1.936(5)<br>min: 1.916(5)<br>mean: 1.925(5)

[a] Max/min/mean values for [**2**]$^{4-}$ and [**3**]$^{4-}$ include both the two {Mo_6} units in each structure, and a disordered {Mo_6} unit in [**2**]$^{4-}$. Esds on mean values are simple averages of the esds of contributing distances. They indicate the average esd on a contributing bond length, not the standard deviation of the mean. Mo^{im} is the imido carrying Mo atom, Mo^t the Mo *trans* to the imido (across the central oxygen), O^c the central oxygen, O^b the oxygens bridging to belt Mo positions to which distances are necessarily averaged. Terminal Mo=O distances in all structures are in the range of 1.65 to 1.70 Å and show no consistent pattern.

3. Cyclic Voltammograms and Differential Pulsed Voltammograms

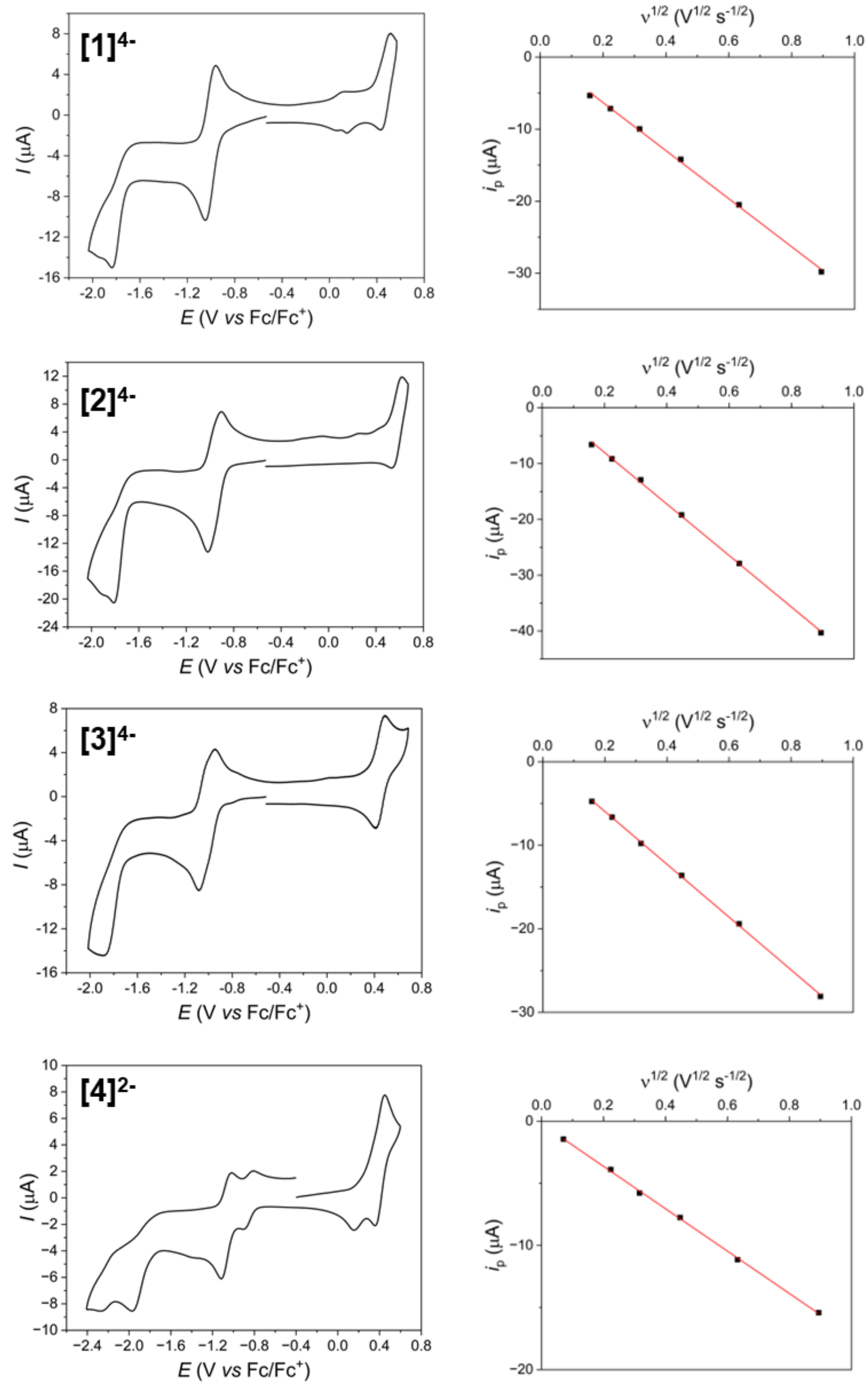

Figure S12 Left: Cyclic voltammograms of $[NBu_4]_4$[**1**] to $[NBu_4]_{42}$[**4**] (bottom) showing aniline oxidations at *ca* 0.45 V, reversible POM reductions at *ca.* -1V and the destructive POM reductions at *ca* -1.8 V (all *vs* Fc/Fc^+). Scan rate 100 mV s^{-1}, GC working electrode, 0.1 M $[NBu_4][BF_4]$ in acetonitrile as electrolyte. Compound $[$**4**$]^{2-}$ shows evidence of a destructive electrochemical process on the oxidation scan, resulting in production of $[Mo_6O_{19}]^{2-}$ and free aniline. **Right:** Peak current *vs* square root of scan rate for the first POM reduction of $[NBu_4]_4$[**1**] (top), $[NBu_4]_4$[**2**] (middle) and $[NBu_4]_4$[**3**] (bottom).

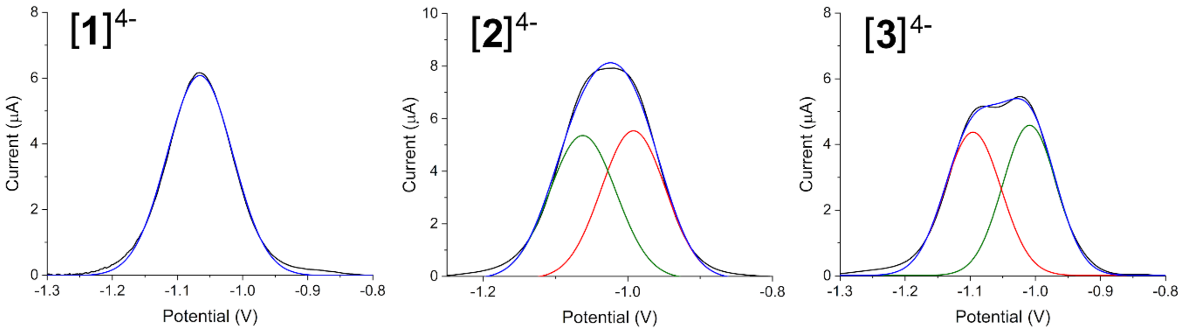

Figure S13: Differential Pulsed Voltammograms of $[NBu_4]_4$[**1**], $[NBu_4]_4$[**2**] and $[NBu_4]_4$[**3**] showing a single two-electron electrochemical process in [**1**]$^{4-}$, and deconvolution of two one-electron processes in [**2**]$^{4-}$ and [**3**]$^{4-}$.

3. Quantum chemistry calculations

Geometry optimizations. Full geometry optimizations were performed at the density functional theory (DFT) level using the ωB97X-D exchange-correlation functional (XCF).[4] This range-separated hybrid functional combines a modified B97 exchange functional[5] for short-range interactions (from 78 to 0%) with HF exchange for long-range interactions (from 22 to 100%), using the default range-separating parameter, ω = 0.2 Bohr^{-1}. Correlation is described by the B97 correlation functional,[5] and empirical atom-atom London dispersion corrections are included as well. For the C, H, N, and O atoms, the atomic basis set consists of 6-311G(d)[6] while LANL2TZ[7] is used for the Mo atoms. TIGHT convergence thresholds on the residual forces on the atoms (1.5 x 10^{-5} Hartree/Bohr or Hartree/radian) were applied. The reliability of the ωB97X-D/6-311G(d)/LANL2TZ method for the geometry optimization of POM derivatives was demonstrated in comparison with other XC functionals in a previous work.[8] To describe the solvent effects (acetonitrile), geometry optimizations were performed in solution using the integral equation formalism (IEF) of the polarizable continuum model (PCM) (IEF-PCM), which represents the solvent by a dielectric continuum characterized by its dielectric permittivity (ε_0 = 35.688 for acetonitrile).[9]

Properties of the excited states. Using the optimized geometries, the time-dependent density functional theory (TD-DFT) method[10] with the same XCF, basis set, and IEF-PCM scheme was used to characterize the (lowest-energy) excited states, considering their vertical excitation energies, $\Delta E_{ge} = E_e - E_g$ (from the ground state g to the excited state e), the corresponding transition dipole moment, μ_{ge}, which is related to the oscillator strength, $f_{ge} = \frac{2}{3}\,\Delta E_{ge}\,\mu_{ge}^2$, and the difference of dipole moment between states g and e, $\Delta\mu_{ge} = \mu_e - \mu_g$. The excited state dipole moments were calculated by using the nonequilibrium IEF-PCM solvation approach (ε_∞ = 1.807 for acetonitrile). Nonequilibrium solvation TDDFT calculations also provided the difference of electron density between the excited and the ground states, $\Delta\rho(\vec{r}) = \rho_e(\vec{r}) - \rho_g(\vec{r})$. Following Le Bahers *et al*,[11] the barycenters of the positive $[\Delta\rho^+(\vec{r})]$ and negative $[\Delta\rho^-(\vec{r})]$ electron density variations were calculated and the distance between them defines the charge-transfer distance (d_{CT}) while the integration over the whole space of $\Delta\rho^+(\vec{r})$ [or

$\Delta\rho^-(\vec{r})$] gives the amount of charge transferred (q_{CT}). The product of these two quantities, $q_{CT}\, d_{CT}$, gives $\Delta\mu_{ge}$. The 30 lowest excitation energies (and oscillator strengths) were calculated.

First hyperpolarizabilities. Using again the same optimized geometries, the SHG β tensor components were evaluated by employing the quadratic response TD-DFT method[12,13] with the same basis set, XCF, and solvation model as for the excited states calculations. Both static and dynamic (incident wavelengths of 1064 nm and 1200 nm) responses were calculated. Computing β of large compounds, including those having donor and/or acceptor substituents, is a challenge for TD-DFT because of the intrinsic nonlocal nature of the response and the approximate XCFs (potential and kernel).[14] However, ωB97X-D is a reliable XCF for calculating the β tensors owing to its substantial amount of long-range HF exchange, as demonstrated in previous investigations where the performance of DFT XCFs was assessed with respect to benchmark wavefunction methods.[15-17] Ref. 8 has also evidenced that using local XCFs or XCFs with small amount of HF exchange leads to overestimated first hyperpolarizabilities. The unit sphere representation (USR) was adopted[18] to visualize the β tensors. First, the induced electric dipole moments

$$\vec{\mu}_{ind} = \overleftrightarrow{\beta} : \vec{E}^2(\theta, \phi) \qquad (1)$$

are evaluated, where $\overleftrightarrow{\beta}$ is the first hyperpolarizability tensor and $\vec{E}(\theta, \phi)$ is a unit vector of electric field, of which the polarization is defined by the θ and ϕ angles (spherical coordinates). Then, the induced dipoles are plotted on a sphere centered on the molecule center of mass. This allows highlighting the directions along which the second-order polarizations are the strongest (i.e. the largest induced dipoles), its orientation (the acceptor-donor direction).

In addition to considering the global β_{HRS} response and its depolarization ratio (ρ), the molecular response has been analyzed by assuming that only a few tensor components are non-zero. The input values are β_{HRS} and ρ while the output values are those tensor components in an effective/ideal Cartesian frame. Assuming Kleinman's conditions are satisfied for a C_{2v} symmetry molecule, there are three independent tensor components, namely β_{zzz}, $\beta_{zyy} = \beta_{yzy} = \beta_{yyz}$, and $\beta_{zxx} = \beta_{xzx} = \beta_{xxz}$. Then, assuming planar symmetry, only two remain, β_{zzz} and $\beta_{zyy} = \beta_{yzy} = \beta_{yyz}$. Their relationships with the observable in VV and HV configurations are, where $\frac{\langle \beta_{ZZZ}^2 \rangle}{\langle \beta_{ZXX}^2 \rangle} = \rho$

$$\langle \beta_{ZZZ}^2 \rangle = \frac{1}{7}{\beta_{zzz}}^2 + \frac{6}{35}\beta_{zzz}\beta_{zyy} + \frac{9}{35}{\beta_{zyy}}^2 \qquad (2)$$

$$\langle \beta_{ZXX}^2 \rangle = \frac{1}{35}{\beta_{zzz}}^2 - \frac{2}{105}\beta_{zzz}\beta_{zyy} + \frac{11}{105}{\beta_{zyy}}^2 \qquad (3)$$

Starting from the macroscopic responses, the β_{zzz} and β_{zyy} quantities have been determined for compounds **1** to **4**. The solutions of the second order equations reported (in the main manuscript) are those that are more realistic given the nature of the molecules and by comparison with the USR, with the sign selected such that the main donor-POM axis points towards negative z, making β_{zzz} positive and β_{zyy} negative. The scheme can also be applied

to molecules such as **3**, even though they differ from C_{2v} symmetry, to obtain *effective* values of β_{zzz} and β_{zyy}.

Computer codes. All DFT and TD-DFT calculations were performed using the Gaussian16 package.[28] The molecular structures, USR pictures, the electron density difference plots, and the simulated UV/vis absorption spectra were generated using the DrawMol software.[29]

Table S3 - Representative bond lengths and bond angles for the compounds in their oxidized forms. The ortho and para positions for compound **3** are defined with respect to the tolyl amine donor group.

	**1**	**2**	**3**		**4**
			ortho	***para***	
C-N (Å)	1.373, 1.373	1.383, 1.378	1.371	1.374	1.371
N-Mo (Å)	1.726, 1.726	1.737, 1.725	1.731	1.726	1.728
C-N-Mo (°)	174.0, 174.4	151.9, 175.0	164.8	178.6	170.8

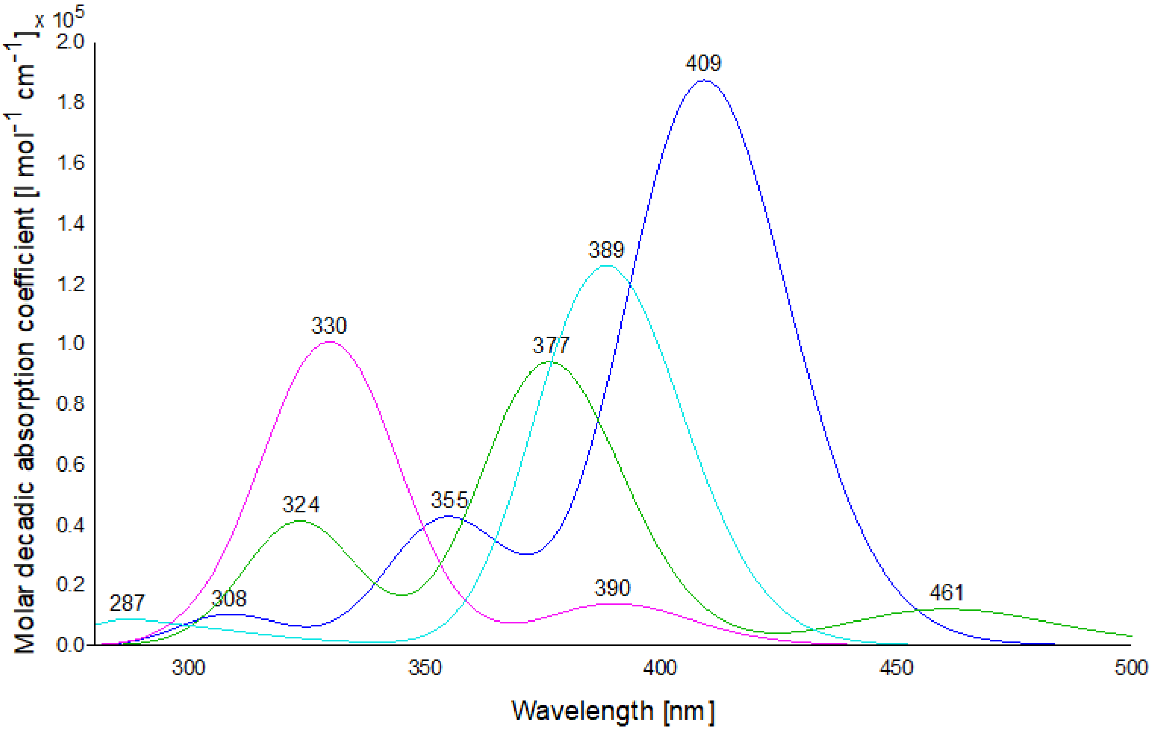

Figure S14. Computed absorption spectra of compounds **1** (blue), **2** (purple), **3** (green) and **4** (cyan) at the IEFPCM(solvent = acetonitrile) TDDFT/ωB97X-D/6-311G(d)/LanL2TZ level of approximation. FWHM = 0.3 eV. 30 excited states have been calculated.

Table S4. Computed charge transfer quantities (q_{CT}), distances (d_{CT}), and dipole moment changes ($\Delta\mu_{ge}$) upon excitation from the GS to the nth dominant lowest-energy ES (μ_{CT}) of **1** to **4** at the IEFPCM(acetonitrile)/TDDFT/wB97X-D/6-311G(d)/LanL2TZ level of approximation. The nonequilibrium solvation approach was adopted.

	n	q_{CT} (e)	d_{CT} (Å)	$\Delta\mu_{ge}$ (D)
Compound **1**	3	0.71	1.71	5.87
	6	0.59	1.39	3.95
Compound **2**	3	1.00	2.53	12.16
	9	0.65	1.84	5.70
	13	0.65	2.44	7.62
Compound **3**	2	1.08	1.99	10.31
	7	0.88	3.42	14.52
	16	0.68	1.85	6.07
Compound **4**	2	0.83	3.47	13.88
	3	0.93	3.35	14.91

References

1. Hsiao, S.-H.; Lin, K.-H. A comparative study on the properties of aromatic polyamides with methyl- or trifluoromethyl-substituted triphenylamine groups. *J. Fluor. Chem.* 2016, **188**, 33-42.

2. (a) Jones, C. F.; Hood, B. R., de Coene, Y.; Lopez-Poves, I.; Champagne, B.; Clays, K.; Fielden, J. Bridge Improvement Work: Maximising Non-Linear Optical Performance in Polyoxometalate Derivatives. *Chem. Commun.* **2024**, *60*, 1731-1734. (b) Wang, K.-L.; Tseng, T.-S.; Tsai, H.-L.; Wu, S.-C. Resistive Switching Polymer Materials based on Poly(arylether)s containing Triphenylamine and 1,2,4-Triazole Moieties. *J. Polym. Sci., Part A: Polym. Chem.* **2008**, *46*, 6861-6871. (c) Novel Aromatic Poly(Amine-Imide)s Bearing a Pendant Triphenylamine Group: Synthesis, Thermal, Photophysical, Electrochemical and Electrochromic Characteristics. *Macromolecules* **2005**, *38*, 307-316. (d) Chang, C.-W.; Chung, C.-H.; Liou, G.-S. Novel Anodic Polyelectrochromic Aromatic Polyamides Containing Pendent Dimethyltriphenylamine Moieties. *Macromolecules* **2008**, *41*, 8441-8451..

3. (a) Al-Yasari, A.; Van Steerteghem, N.; El Moll, H.; Clays, K.; Fielden, J. Donor–Acceptor Organo-Imido Polyoxometalates: High Transparency, High Activity Redox-Active NLO Chromophores. *Dalton Trans*. **2016**, *45*, 2818-2822. (b) Al-Yasari, A.; Van Steerteghem, N.; Kearns, H.; El Moll, H.; Faulds, K.; Wright, J. A.; Brunschwig, B. S.; Clays, K.; Fielden, J. Organoimido-Polyoxometalate Nonlinear Optical Chromophores: A Structural, Spectroscopic, and Computational Study. *Inorg. Chem.* **2017**, *17*, 10181-10194. (c) Strong, J. B.; Yap, G. P. A.: Ostrander, R.; Liable-Sands, L. M.; Rheingold, A. L.; Thouvenot, R.; Gouzerh, P.; Maatta. E. A. A New Class of Functionalized Polyoxometalates: Synthetic, Structural, Spectroscopic, and Electrochemical Studies of Organoimido Derivatives of $[Mo_6O_{19}]^{2-}$. *J. Am. Chem. Soc.* **2000**, *122*, 639-649. (d) Xu, B.; Wei, Y.; Barnes, C. L.; Peng, Z. Hybrid Molecular Materials Based on Covalently Linked Inorganic Polyoxometalates and Organic Conjugated Systems. *Angew. Chem. Int. Ed.* **2001**, *40*, 2290-2292.

4. Chai, J. D.; Head-Gordon, M. Long-Range Corrected Hybrid Density Functionals with Damped Atom-Atom Dispersion Corrections. *Phys. Chem. Chem. Phys.* **2008**, *10*, 6615-6620.

5. Becke, A. D. Density-Functional Thermochemistry. V. Systematic Optimization of Exchange Correlation Functionals. *J. Chem. Phys*. **1997**, *107*, 8554-8560

6. Krishnan, R.; Binkley, J. S.; Seeger, R.; Pople, J. A. Self-consistent Molecular Orbital Methods. XX. A Basis Set for Correlated Wavefunctions. *J. Chem. Phys.* **1980**, *72*, 650-654.

7. Roy, L.E.; Hay, P. J.; Martin, R. L. Revised Basis Sets for the LANL Effective Core Potentials. *J. Chem. Theory Comput.* **2008**, *4*, 1029-1031

8. Rtibi, E.; Abderrabba, M.; Ayadi, S.; Champagne, B. Theoretical Assessment of the Second-Order Nonlinear Optical Responses of Lindqvist-Type Organoimido Polyoxometalates. *Inorg. Chem*. **2019**, *58*, 11210-11219.

9. Tomasi, J.; Mennucci, B.; Cammi, R. Quantum Mechanical Continuum Solvation Models. *Chem. Rev*. **2005**, *105*, 2999-3094.

10. Casida, M. E.; pp 155-192 in *Recent advances in Density Functional Theory*, ed. Chong, D. P.; World Scientific: Singapore, **1995**.

11. Le Bahers, T.; Adamo, C.; Ciofini, I. A Qualitative Index of Spatial Extent in Charge-Transfer Excitations. *J. Chem. Theory Comput.* **2011**, *7*, 2498-2506.

12. Van Gisbergen, S. J. A.; Snijders, J. G.; Baerends, E. J. Calculating Frequency-Dependent Hyperpolarizabilities using Time-Dependent Density Functional Theory. *J. Chem. Phys*. **1998**, *109*, 10644-10656.

13. Helgaker, T.; Coriani, S.; Jørgensen, P.; Kristensen, K.; Olsen, J.; Ruud, K. Recent Advances in Wave Function-Based Methods of Molecular Property Calculations. *Chem. Rev.* **2012**, *112*, 543-631.

14. Champagne, B.; Beaujean, P.; de Wergifosse, M.; Cardenuto, M.; Liégeois, V.; Castet, F. pp 117-138 in *Frontiers of Quantum Chemistry*, Eds: Wojcik, M.; Nakatsuji, H.; Kirtman, B.; Ozaki, Y.; Springer: Singapore, **2018**.

15. de Wergifosse, M.; Champagne, B. Electron Correlation Effects on the First Hyperpolarizability of Push-Pull π-Conjugated Systems. *J. Chem. Phys.* **2011**, *134*, 074113.

16. Johnson, L. E.; Dalton, L. R.; Robinson, B. H. Optimising Calculations of Electronic Excitations and Relative Hyperpolarizabilities of Electrooptic Chromophores. *Acc. Chem. Res*. **2014**, *47*, 3258-3265.

17. Garrett, K.; Sosa Vazquez, X. A.; Egri, S. B.; Wilmer, J.; Johnson, L. E.; Robinson, B. H.; Isborn, C. M. Optimum Exchange for Calculation of Excitation Energies and Hyperpolarizabilities of Organic Electro-optic Chromophores, *J. Chem. Theory Comput.* **2014**, *10*, 3821-3831.

18. Tuer, A.; Krouglov, S.; Cisek, R.; Tokarz, D.; Barzda, V. Three-Dimensional Visualization of the First Hyperpolarizability Tensor. *J. Comput. Chem.* **2011**, *32*, 1128-1134.

19. Campo, J.; Wenseleers, W.; Goovaerts, E.; Szablewski, M.; Cross. G. H. Accurate Determination and Modelling of the Dispersion of the First Hyperpolarizability of an Efficient Zwitterionic Nonlinear Optical Chromophore by Tunable Wavelength Hyper-Rayleigh Scattering. *J. Phys. Chem. C* **2008,** *112*, 287-296.

20. de Wergifosse, M. ; Botek, E.; De Meulenaere, E.; Clays, K.; Champagne, B. ONIOM Investigation of the Second-Order Nonlinear Responses of Fluorescent Proteins. *J. Phys. Chem. B* **2018**, *122*, 4993-5005.

28. Frisch, M. J.; Trucks, G. W.; Schlegel, H. B.; Scuseria, G. E.; Robb, M. A.; Cheeseman, J. R.; Scalmani, G.; Barone, V.; Petersson, G. A.; Nakatsuji, H.; Li, X.; Caricato, M.; Marenich, A. V.; Bloino, J.; Janesko, B. G.; Gomperts, R.; Mennucci, B.; Hratchian, H. P.; Ortiz, J. V.; Izmaylov, A. F.; Sonnenberg, J. L.; Williams-Young, D.; Ding, F.; Lipparini, F.; Egidi, F.; Goings, J.; Peng, B.; Petrone, A.; Henderson, T.; Ranasinghe, D.; Zakrzewski, V. G.; Gao, J.; Rega, N.; Zheng, G.; Liang, W.; Hada, M.; Ehara, M.; Toyota, K.; Fukuda, R.; Hasegawa, J.; Ishida, M.; Nakajima, T.; Honda, Y.; Kitao, O.; Nakai, H.; Vreven, T.; Throssell, K.; Montgomery Jr., J. A.; Peralta, J. E.; Ogliaro, F.; Bearpark, M. J.; Heyd, J. J.; Brothers, E. N.; Kudin, K. N.; Staroverov, V. N.; Keith, T. A.; Kobayashi, R.; Normand, J; Raghavachari, K.; Rendell, A. P.; Burant, J. C.; Iyengar, S. S.; Tomasi, J.; Cossi, M.; Millam, J. M.; Klene, M.; Adamo, C.; Cammi, R.; Ochterski, J. W.; Martin, R. L.; Morokuma, K.; Farkas, O.; Foresman, J. B.; Fox, D. J. Gaussian16 Revision A.03, **2016**.

29. DrawMol, Liégeois, V. U. Namur, www.unamur.be/drawmol.
